# Polyketides from a Marine-Derived Fungus *Xylariaceae* sp.

**DOI:** 10.3390/md11051718

**Published:** 2013-05-21

**Authors:** Xu-Hua Nong, Zhi-Hui Zheng, Xiao-Yong Zhang, Xin-Hua Lu, Shu-Hua Qi

**Affiliations:** 1 Key Laboratory of Marine Bio-Resources Sustainable Utilization, Guangdong Key Laboratory of Marine Materia Medica, RNAM Center for Marine Microbiology, South China Sea Institute of Oceanology, Chinese Academy of Sciences, 164 West Xingang Road, Guangzhou 510301, Guangdong, China; E-Mails: nongxuhua4883@163.com (X.-H.N.); zhangxiaoyong@scsio.ac.cn (X.-Y.Z.); 2 University of Chinese Academy of Sciences, Beijing 100049, China; 3 New Drug Research & Development Center of North China Pharmaceutical Group Corporation, Shijiazhuang, Hebei 052260, China; E-Mails: z_zhihui2003@yahoo.com.cn (Z.-H.Z.); luxinhua@ncpcrd.com.cn (X.-H.L.)

**Keywords:** *Xylariaceae *sp., polyketide, enzyme-inhibitory activity, antifouling activity

## Abstract

Eighteen polyketides (**1**–**18**) including six citrinin derivatives, two phenol derivatives, one cyclopentenone, two naphthol derivatives, and seven tetralone derivatives were isolated from the culture broth of a marine-derived fungal strain *Xylariaceae *sp. SCSGAF0086. Five of these compounds (**1**, **2**, **8**, **9**, and **10**) were new, and their structures were determined by spectroscopic methods. Compounds **4**, **6**, **7**, and **17** showed enzyme-inhibitory activities towards several tested enzymes, and **6** and **7** showed strong antifouling activity against *Bugula neritina *larvae settlement. This is the first time that the antifouling and enzyme-inhibitory activities of these compounds has been reported.

## 1. Introduction

**Figure 1:**
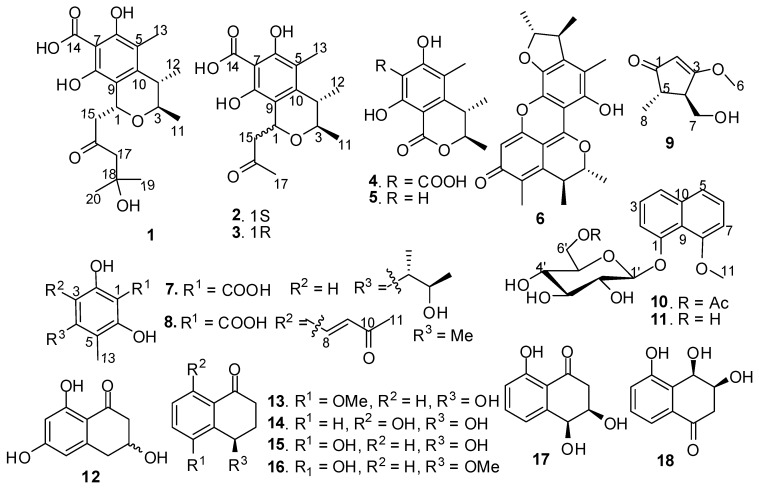
Structures of compounds **1**–**18**.

Gorgonian surfaces are frequently colonized by diverse fungi. These symbiotic fungi are becoming new sources of various bioactive compounds [1,2,3,4,5]. During the course of our investigation on secondary metabolites of marine gorgonian-derived fungi, the fungal strain *Xylariaceae *sp. SCSGAF0086 attracted our attention. Preliminary experiment showed that the culture extract of the strain had antifouling and antibacterial activities. Further investigation on the chemical constituents of the extract led to the obtainment of 18 polyketides (**1**–**18**) (Figure 1) from *Xylariaceae *sp. SCSGAF0086. These polyketides included six citrinin derivatives, two phenol derivatives, one cyclopentenone, two naphthol derivatives, and seven tetralone derivatives, which displayed the diversity of secondary metabolites of the marine-derived fungal strain. Five of these compounds (**1**, **2**, **8**, **9**, and **10**) were new, and the other compounds were identified as 1-acetonyl-7-carboxyl-6,8-dihydroxy-3,4,5-trimethylisochroman (**3**) [6], dihydrocitrinone (**4**) [7], stoloniferol B (**5**) [7], dicitrinin A (Wakana named it penicitrinone A, **6**) [8,9], phenol A acid (**7**) [9], 8-methoxy-1-naphthyl β-glucopyranoside (**11**) [10], (±)-scytalone (**12**) [11], (4*R*)-(−)-5-*O*-methylsclerone (**13**) [12], (4*R*)-(−)-4,8-dihydroxy-1-tetralone (**14**) [13,14], (4*R*)-(−)-regiolone (**15**) [15], (4*R*)-(−)-5-hydroxy-4-methoxy-1-tetralone (**16**) [16], (3*R*,4*S*)-(+)-4-hydroxy-6-deoxyscytalone (**17**) [17], and (3*S*,4*R*)-(+)-3,4,5-trihydroxy-1-tetralone (**18**) [18].

Compounds **1**–**18** were tested for enzyme-inhibitory activities towards cathepsin B, inosine monophosphate dehydrogenase (IMPDH), protein tyrosine phosphatase 1B (PTPlB), and Src homology 2 domain-containing phosphotyrosine phosphatase (SHP2), and antifouling activity toward *Bugula neritina *larvae settlement. This paper describes the isolation, structure elucidation, enzyme-inhibitory and antifouling activities of these compounds.

## 2. Results and Discussion

Compound **1** was obtained as turquoise amorphous solid. Its molecular formula of C_19_H_26_O_7_ was determined by HRESIMS (*m*/*z* 389.1592 [M + Na]^+^). The ^1^H NMR spectrum exhibited five methyl signals at δ_H_ 1.01 (d, *J* = 6.5 Hz, Me-11), 1.15 (s, Me-19), 1.16 (s, Me-20), 1.18 (d, *J* = 6.5 Hz, Me-12), 1.92 (s, Me-13), and two oxymethine protons at δ_H_ 3.85 (qd, *J* = 2.0, 6.5 Hz, H-3), 5.07 (dd, *J* = 2.0, 9.5 Hz, H-1). The ^13^C NMR and DEPT spectra revealed the presence of 19 carbon signals including five methyls [δ_C_ 9.5 (Me-13), 18.2 (Me-11), 19.9 (Me-12), 29.4 (overlapped, Me-19 and Me-20)], two methylenes [δ_C_ 49.8 (C-15), 55.5 (C-17)], three methines [δ_C_ 35.0 (C-4), 65.3 (C-1), 71.5 (C-3)], one oxyquaternary carbon (δ_C_ 68.5, C-18), one fully substituted phenyl ring [δ_C_ 101.7 (s, C-7), 109.4 (s, C-5), 110.6 (s, C-9), 139.5 (s, C-10), 155.7 (s, C-8), 158.4 (s, C-6)], one carboxyl group (δ_C_ 175.5, C-14), and one carbonyl group (δ_C_ 209.0, C-16). These data showed close similarity to those of **3** [6], which suggested that **1** had a citrinin skeleton. Comparison of the NMR data of **1** and **3** showed that the obvious difference between them was the additional appearance of one methyl (δ_C_ 29.4), one methylene (δ_C_ 55.5), and one oxyquaternary carbon (δ_C_ 68.5) in **1**, which indicated that the only difference between **1** and **3** was the side chain substituted at C-1.

**Figure 2:**
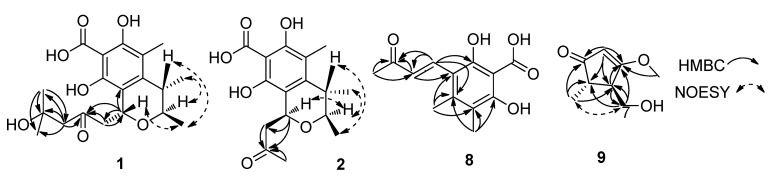
Key HMBC and NOESY correlations of **1**, **2**, **8** and **9**.

This was proved by the HMBC spectrum of **1** (Figure 2). The HMBC correlations of Me-19 with C-17/C-18/C-20, Me-20 with C-17/C-18/C-19, H-17 with C-16/C-18/C-19/C-20, H-15 with C-1/C-16, and H-1 with C-9/C-15/C-16, suggested the attachment of a (CH_3_)_2_C(OH)–CH_2_–CO–CH_2_– group at C-1. The relative configuration of **1** was further determined by NOESY spectrum (Figure 2). The NOE correlation of H-3 with Me-12 suggested the α-configuration of H-3 and Me-12, while the NOE correlations of Me-11 with H-1/H-4 suggested that H-1, H-4 and Me-11 possesed β-configuration. Its relative configuration was identical to that of compound **3** according to their identical NOE data. The specific optical rotation value of **1** ([α]^2^_D_ 24.8 (*c* 0.08, MeOH)) was also similar to that of **3** ([α]^2^_D_ 59.2 (*c* 0.37, MeOH)), which suggested that the absolute configuration of **1** was 1*R*,3*R*,4*S*. So, the structure of **1** was elucidated as shown and named penicitrinol F.

Compound **2** has the same molecular formula of C_16_H_20_O_6_ as **3**, which was inferred from HRESIMS (*m*/*z* 331.1171 [M + Na]^+^). The ^1^H and ^13^C NMR spectroscopic data of **2** showed great similarity to those of **3** [6], except the obvious chemical shift changes of two signals [δ_C_ 68.5 (d, C-1), 73.7 (d, C-3) in **3**, and correspondingly δ_C_ 65.5 (d, C-1), 71.7 (d, C-3) in **2**]. Further detailed analysis of HSQC and HMBC spectra (Figure 2) suggested that **2** should have the same plane structure as **3**, and it might be an isomer of **3** at C-1. This was supported by the NOESY spectrum of **2** (Figure 2). The NOE correlations of H-3 with H-1/Me-12 indicated that H-1, H-3, and Me-12 possessed α-configuration, while the NOE correlation of H-4 with Me-11 suggested that H-4 and Me-11 possessed β-configuration. By comparison with that of **3**, the absolute configuration of **2** was deduced as 1*R*,3*R*,4*S*. Therefore, the structure of **2** was determined as shown. Because the only structural difference between **2** and penicitrinol C [19] was the additional substituent of a carboxyl group at C-7 in **2**, so **2** was named 7-carboxypenicitrinol C. 

Compound **8** has a molecular formula of C_13_H_14_O_5_ as inferred from HRESIMS (*m*/*z* 251.0666 [M + H]^+^). The ^1^H NMR spectrum showed three methyls at δ_H_ 2.00 (s, Me-13), 2.23 (s, Me-11), 2.27 (s, Me-12), two olefin protons at δ_H_ 7.02 (d, *J* = 16.0 Hz, H-9), 7.77 (d, *J* = 16.0 Hz, H-8) and two hydroxyl groups at δ_H_ 15.70 (2-OH), 16.23 (6-OH). The ^13^C NMR spectrum showed the presence of three methyls [δ_C_ 11.2 (Me-13), 16.6 (Me-12), 27.5 (Me-11)], six aromatic quaternary carbons [δ_C_ 101.3 (C-1), 109.8 (C-3), 112.7 (C-5), 141.1 (C-4), 162.5 (C-2), 162.6 (C-6)], one double bond [δ_C_ 125.5 (d, C-9), 138.6 (d, C-8)], one carboxyl carbon (δ_C_ 175.5, C-7), and one carbonyl carbon (δ_C_ 198.3, C-10). These NMR data showed similarity to those of **7** [9], which suggested that **8** was a fully substituted benzoic acid derivative. In the HMBC spectrum (Figure 2), the correlations of Me-11 with C-9/C-10, H-8 with C-2/C-4/C-9/C-10, and H-9 with C-3/C-10, suggested a CH_3_–CO–CH=CH– group attached at C-3 of the aromatic ring. A large coupling constant between H-8 and H-9 (*J*_8,9_ = 16.0 Hz) indicated *E*-configuration of the double bond. Furthermore, the HMBC correlations of Me-13 with C-4/C-5/C-6, and Me-12 with C-3/C-4/C-5, suggested two methyl groups attached at C-4 and C-5, respectively. Based on the above data, the structure of **8** was determined as shown and named 2, 6-dihydroxy-4,5-dimethyl-3-(3-oxo-1-butenyl)benzoic acid.

Compound **9** has a molecular formula of C_8_H_12_O_3_ as inferred from HRESIMS (*m*/*z* 157.0854 [M + H]^+^). The ^1^H NMR spectrum exhibited one methyl at δ_H_ 1.06 (d, *J* = 7.5 Hz, H-8), two methines at δ_H_ 2.32 (qd, *J* = 7.5, 3.0 Hz, H-5), 2.47 (ddd, *J* = 1.0, 3.0, 6.5 Hz, H-4), one oxymethylene at δ_H_ 3.61 (m, H-7), one oxymethyl at δ_H_ 3.80 (s, H-6) and one olefinic methine at δ_H_ 5.32 (s, H-2). The ^13^C NMR and DEPT spectra revealed the presence of eight carbons including one methyl (δ_C_ 15.3, Me-8), two methines [δ_C_ 42.8 (C-4), 50.9 (C-5)], one oxymethyl (δ_C_ 58.7, C-6), one oxymethylene (δ_C_ 59.5, C-7), one double bond [δ_C_ 103.4 (d, C-2), 188.8 (s, C-3)], and one carbonyl group (δ_C_ 205.9, C-1). Furthermore, the HMBC spectrum (Figure 2) showed that the correlations of H-8 with C-1/C-4/C-5, H-7 with C-3/C-4/C-5, H-6 with C-3, H-2 with C-1/C-3/C-4/C-5, and H-4 with C-3/C-7, which established the plane structure of **9**. The relative configuration of **9** was further assigned by NOESY spectrum. The NOE correlation of H-7 with H-5 suggested that protons H-4 and H-5 were on the different side, which was supported by the coupling constant between H-4 and H-5 (*J*_4,5_ = 3.0 Hz). Based on the above data, the structure of **9** was determined as shown and named 4-(hydroxymethyl)-3-methoxy-5-methylcyclopent-2-enone. It is interesting that **9** has the same cyclopent-2-enone skeleton as cyclopentenone prostaglandins those are commonly present in many species of gorgonians [20].

Compound **10** has a molecular formula of C_19_H_22_O_8_ as inferred from HRESIMS (*m*/*z* 401.1209 [M + Na]^+^). The ^1^H NMR spectrum exhibited signals for one methyl at δ_H_ 2.17 (s), seven sugar protons at δ_H_ 3.59–4.81 [3.59 (1H, m, H-2′), 3.71 (1H, m, H-4′), 3.72 (1H, m, H-5′), 3.82 (1H, t, *J* = 9.5, H-3′), 4.44 (1H, dd, *J* = 1.5, 12.5 Hz, H-6′a), 4.62 (1H, dd, *J* = 4.5, 12.5 Hz, H-6′b), 4.81 (1H, d, *J* = 7.0 Hz, H-1′)], one oxymethyl at δ_H_ 4.00 (s, Me-11), and six protons of aromatic ABC and A′B′C′ systems at δ_H _6.96 (1H, d, *J* = 8.0 Hz, H-2), 7.36 (1H, br t, *J* = 8.0 Hz, H-3), 7.38 (1H, br t, *J *= 8.0 Hz, H-6), 7.41 (1H, dd, *J* = 2.5, 8.0 Hz, H-4), 7.48 (1H, d, *J* = 8.0 Hz, H-7), 7.58 (1H, dd, *J* = 2.5, 8.0 Hz, H-5). The ^13^C NMR and DEPT spectra revealed the presence of 19 carbon signals including one methyl (δ_C_ 20.9, Me-13), one oxymethyl (δ_C_ 57.2, Me-11), a sugar moiety [δ_C_ 63.3 (t, C-6′), 69.9 (d, C-4′), 74.0 (d, C-3′), 74.5 (d, C-5′), 75.8 (d, C-2′), 105.1 (d, C-1′)], ten aromatic carbons [δ_C_ 108.5 (d, C-2), 114.7 (d, C-7), 118.4 (s, C-9), 122.1 (d, C-4), 124.2 (d, C-5), 126.4 (d, C-6), 126.5 (d, C-3), 136.2 (s, C-10), 155.0 (s, C-1), 155.6 (s, C-8)], and one carboxyl group [δ_C_ 171.6 (COOCH_3_)]. These NMR data showed remarkable similarity to those of **11** [10], and the only difference between **10** and **11** was the additional presence of one acetyl group in **10**, which suggested that **10** had the same structural skeleton as **11** and the sugar moiety was glucose [10,21]. In the HMBC spectrum, the correlations of H-6′ with C-12, and Me-13 with C-12 suggested the acetylation of 6′-OH of the glucose moiety. The large coupling constant of 7.0 Hz for the anomeric proton of the glucose moiety (δ_H_ 4.81) suggested that the glucose was β-glucose [10,21,22]. Therefore, the structure of **10** was determined as shown and named 8-methoxy-1-naphthyl 6′-*O*-acetyl-β-glucopyranoside. 

Compounds **1**–**18** were tested for their inhibitory activities towards enzymes of cathepsin B, IMPDH, PTPlB and SHP2. The results displayed that **4** and **7** had inhibitory activities towards cathepsin B with IC_50_ values of 28.5 ± 1.7, 20.4 ± 1.9 μM, respectively, **6** showed inhibitory activities towards SHP2 and IMPDH with IC_50_ values of 58.4 ± 4.0 and 98.7 ± 6.2 μM, respectively, and **17** showed inhibitory activities towards SHP2, PTPlB, and IMPDH with IC_50_ values of 4.1 ± 0.6, 13.9 ± 1.8, and 41.2 ± 3.6 μM, respectively, while other compounds showed very weak or no activity towards all tested enzymes. The positive controls used in cathepsin B and IMPDH enzyme-inhibitory assays were leupeptin and mycophenolic acid, with IC_50_ values of 0.03 and 0.4 μM, respectively. Similarly, the ursolic acid used as positive controls in PTPlB and SHP2 enzyme-inhibitory assays showed an identical IC_50_ value of 2.8 μM. Cathepsin B and SHP2 are targets for screening anti-tumor agents, and IMPDH and PTPlB are targets for screening immunosuppressive and anti-diabetic agents, respectively. Previous studies reported that **17** was a phytotoxic substance and an important melanin biosynthetic intermediate in fungi [23]. Moreover, **17** was found to be a SHP2 and PTPlB inhibitor. This is the first time that the enzyme-inhibitory activities of these compounds has been reported.

In addition, antifouling activities of compounds **1**–**18** were tested against larvae settlement of *B. neritina *larva. The results showed that **6** and **7** had strong antifouling activity against *B. neritina *larvae settlement with EC_50_ values of 1.76 ± 0.8 and 14.35 ± 1.72 μg/mL, and LC_50_/EC_50_ values of >56, and >15, respectively, while other compounds showed weak or no activity. Usually, the standard requirement of an efficacy EC_50_ level for natural antifoulant is 25 μg/mL, and an antifouling compound with LC_50_/EC_50_ >15 is often considered as a non-toxic antifouling compound [24]. The results indicated that **6** and **7** were potential natural antifouling candidates. This is the first time that the antifouling activities of these compounds has been reported.

## 3. Experimental Section

### 3.1. General Experimental Procedures

Optical rotations were measured with a PL341 spectropolarimeter. UV spectra were measured with a Shimadzu UV-2501PC spectrophotometer in MeOH solution. IR spectra were measured with a FT-IR NICOLET spectrophotometer. ^1^H, ^13^C NMR and 2D NMR spectra were recorded on a Bruker AV-500 MHz NMR spectrometer with TMS as reference. MS spectroscopic data were obtained on a LCQDECA XP HPLC/MSn spectrometer for ESIMS. High-resolution electrospray-ionization (HRESIMS) was performed on a UPLC/Q-TOF Micro MS spectrometer under 70 eV. Semi-preparative HPLC was performed on a Shimadzu LC-20A preparative liquid chromatography with an YMC-Pack ODS column, 250 × 10 mm i.d., S-5 μm. Sephadex LH-20 (GE Healthcare) was used for CC. Silica gel (200–300 mesh) for CC and GF254 for TLC were obtained from the Qindao Marine Chemical Factory, Qindao, China.

### 3.2. Fungal Material

The fungal strain SCSGAF0086 (GenBank access number JN851017) was isolated from the South China Sea gorgonian corals *Melitodes squamata*, and identified as *Xylariaceae* sp. SCSGAF0086 by a molecular biological protocol calling for DNA amplification and ITS region sequence comparison with GenBank database, sharing a similarity of 89% with *Xylariaceae* sp., which was deposited in RNAM center, South China Sea Institute of Oceanology, Chinese Academy of Sciences.

### 3.3. Fermentation and Extraction

The strain was inoculated in PDA liquid medium (containing 20 g/L glucose, 200 g/L potato and 30 g/L sea salt) in 500 mL shake flask loading 120 mL as seed culture and incubated on a rotary shaker (200 rpm) at 28 °C for 3 days. Then, 20 L broth inoculated with seed culture as scale-up cultivation were carried out under static condition at 28 °C for 50 days in 120 of 500-mL conical flasks containing the liquid medium (150 mL/flask) composed of glucose (10 g/L), maltose (20 g/L), mannitol (20 g/L), monosodium glutamate (10 g/L), KH_2_PO_4_ (0.5 g/L), MgSO_4_·7H_2_O (0.3 g/L), corn steep liquor (0.5 g/L), yeast extract (3 g/L), and seawater (adjusted to pH 7.0 with 1 mol/L hydrochloric acid before sterilization).

After incubation, the 20 L fermentation broth was filtered through cheesecloth to separate the broth supernatant and mycelia, respectively. For fermentation broth, the supernatant was extracted with ethyl acetate while mycelia was extracted with 80% acetone; the acetone extract was evaporated under reduced pressure to afford an aqueous solution, and extracted with ethyl acetate; The extracts of filtrate and mycelium were concentrated *in vacuo* to give a crude residue.

### 3.4. Isolation and Purification

The residue (10.5 g) was subjected to a reversed phase RP-18 CC eluting with MeOH-H_2_O solvent system at the ratios of 100:0, 80:20, 65:35, 45:55, 25:75, 0:100 (v/v) to yield 11 fractions. Fraction 3 (234 mg) was subjected to repeated Sephadex LH-20 CC eluting with MeOH to obtain **18** (2.3 mg). Fraction 4 (640 mg) was fractionated by Sephadex LH-20 CC eluting with MeOH to offer six subfractions (4-1–4-6), and then subfraction 4-1 was purified by semi-preparative reversed-phase (SP-RP) HPLC (YMC-Pack, ODS S-5 μ 250 × 10 mm i.d., 3 mL/min) eluting with MeOH-H_2_O (20:80) to give **9** (1.2 mg), further purification of subfraction 4-2 was achieved by preparative TLC with a mobile phase of CHCl_3_-MeOH (8:2) to give **12** (5.3 mg), **15** (3.3 mg), and **17** (20.3 mg), subfraction 4-4 was purified by a silica gel CC eluting with CHCl_3_-MeOH at the ratios of 95:5, 90:10, 80:20, 70:30 (v:v), to offer **7** (4.6 mg), and subfraction 4-6 was purified by SP-RP-HPLC with MeOH-H_2_O-TFA (30:70:0.03) to obtain **4** (8.9 mg). Fraction 5 (260 mg) was separated by Sephadex LH-20 CC to afford seven subfractions. Subfraction 5-5 was purified by SP-RP-HPLC eluting with MeOH-H_2_O (30:70) to obtain **13** (8.2 mg) and **14** (6.9 mg). Subfraction 5-6 was subjected to Sephadex LH-20 CC to give a mixture of isomers **2** and **3**, and the mixture was further purified by SP-RP-HPLC, eluting with MeOH-H_2_O-TFA (47:53:0.03) to afford **2** (2.8 mg, *t_R_* = 16 min) and **3** (4.8 mg, *t_R_* = 20 min). Subfraction 5-7 was purified by repeated Sephadex LH-20 CC to obtain **8** (2.9 mg). Fraction 6 (100 mg) was subjected to repeated Sephadex LH-20 CC eluting with MeOH to obtain **1** (3.5 mg), and **16** (7.4 mg). Fraction 7 (460 mg) was fractionated by Sephadex LH-20 CC, then further purified by SP-RP-HPLC using MeOH-H_2_O (45:55) to give **5** (2.3 mg) and **11** (4.3 mg). Fraction 8 (565 mg) was fractionated by repeated Sephadex LH-20 CC eluting with MeOH to give **10** (2.9 mg). Fraction 9 (410 mg) was subjected to Sephadex LH-20 CC, then purified by SP-RP-HPLC eluting with MeOH-H_2_O (70:30) to afford **6** (2.3 mg).

Penicitrinol F (**1**): Turquoise amorphous solid; [α]^2^_D_ 24.8 (*c* 0.08, MeOH); UV (MeOH) λ_max_ (log ε): 383(2.15) nm; IR (KBr) ν_max_: 3417, 2975, 2932, 1686, 1626, 1595, 1497, 1427, 1407, 1382, 1208 cm^−1^; ^1^H-NMR (500 MHz, DMSO-*d*_6_) δ_H_: 15.18 (1H, br s, 8-OH), 14.67 (1H, br s, 6-OH), 5.07 (1H, dd, *J *= 2.0, 9.5 Hz, H-1), 4.47 (1H, br s, 18-OH), 3.85 (1H, qd, *J *= 2.0, 6.5 Hz, H-3), 3.23 (1H, dd, *J* = 2.0, 15.3 Hz, CH_2_-15), 2.63 (1H, dd, *J* = 9.5, 15.3 Hz, CH_2_-15), 2.56 (2H, s, CH_2_-17), 2.53 (1H, qd, *J *= 2.0, 6.5 Hz, H-4), 1.92 (3H, s, Me-13), 1.18 (3H, d, *J *= 6.5 Hz, Me-12), 1.16 (3H, s, Me-20), 1.15 (3H, s, Me-19), 1.01 (3H, d, *J *= 6.5 Hz, Me-11); ^13^C-NMR (125 MHz, DMSO-*d*_6_) δ_C_: 209.0 (C, C-16), 175.5 (C, C-14), 158.4 (C, C-6), 155.7 (C, C-8), 139.5 (C, C-10), 110.6 (C, C-9), 109.4 (C, C-5), 101.7 (C, C-7), 71.5 (CH, C-3), 68.5 (C, C-18), 65.3 (CH, C-1), 55.5 (CH_2_, C-17), 49.8 (CH_2_, C-15), 35.0 (CH, C-4), 29.4 (CH_3_, overlapped, C-19 and C-20), 19.9 (CH_3_, C-12), 18.2 (CH_3_, C-11), 9.5 (CH_3_, C-13); HRESIMS *m*/*z* 389.1592 [M + Na]^+^ (calcd. for C_19_H_26_NaO_7_, 389.1576).

7-Carboxypenicitrinol C (**2**): Yellow amorphous solid; [α]^2^_D_ −31.7 (*c *0.42, MeOH); UV (MeOH) λ_max_ (log ε): 215(3.15), 252(2.65), 320(2.19) nm; IR (KBr) ν_max_: 3427, 2973, 2931, 2832, 2718, 1698, 1591, 1418, 1364, 1264 cm^−1^; ^1^H-NMR (500 MHz, DMSO-*d*_6_) δ_H_: 14.91 (1H, br s, 8-OH), 14.41 (1H, br s, 6-OH), 5.08 (1H, dd, *J *= 3.0, 9.5 Hz, H-1), 3.87 (1H, qd, *J* = 1.0, 6.5 Hz, H-3), 3.44 (1H, dd, *J* = 3.0, 14.7 Hz, H-15a), 2.55 (1H, qd, *J* = 1.0, 7.0 Hz, H-4), 2.48 (1H, dd, *J* = 9.5, 14.7 Hz, H-15b), 2.12 (3H, s, Me-17), 1.93 (3H, s, Me-13), 1.19 (3H, d, *J *= 7.0 Hz, Me-12), 1.03 (3H, d, *J* = 6.5 Hz, Me-11); ^13^C-NMR (125 MHz, DMSO-*d*_6_) δ_C_: 207.4 (C, C-16), 175.8 (C, C-14), 158.4 (C, C-6), 155.6 (C, C-8), 140.0 (C, C-10), 110.7 (C, C-9), 109.8 (C, C-5), 101.4 (C, C-7), 71.7 (CH, C-3), 65.5 (CH, C-1), 49.1 (CH_2_, C-15), 35.1 (CH, C-4), 30.0 (CH_3_, C-17), 20.0 (CH_3_, C-11), 18.2 (CH_3_, C-12), 9.5 (CH_3_, C-13); HRESIMS *m*/*z* 331.1171 [M + Na]^+^ (calcd. for C_16_H_20_NaO_6_, 331.1158).

2,6-Dihydroxy-4,5-dimethyl-3-(3-oxo-1-butenyl) benzoic acid (**8**): Yellow amorphous solid; UV (MeOH) λ_max_ (log ε): 214(2.50), 249(2.43), 372(2.37) nm; IR (KBr) ν_max_: 3434, 2922, 1581, 1406, 1364, 1256, 1497, 1228, 1155 cm^−1^; ^1^H-NMR (500 MHz, DMSO-*d*_6_) δ_H_: 16.23 (1H, s, 2-OH), 15.70 (1H, s, 6-OH), 7.77 (1H, d, *J* = 16.0 Hz, H-8), 7.02 (1H, d, *J* = 16.0 Hz, H-9), 2.27 (3H, s, Me-12), 2.23 (3H, s, Me-11), 2.00 (3H, s, Me-13); ^13^C-NMR (125 MHz, DMSO-*d*_6_) δ_C_: 198.3 (C, C-10), 175.5 (C, C-7), 162.6 (C, C-6), 162.5 (C, C-2), 141.1 (C, C-4), 138.6 (CH, C-8), 125.5 (CH, C-9), 112.7 (C, C-5), 109.8 (C, C-3), 101.3 (C, C-1), 27.5 (CH_3_, C-11), 16.6 (CH_3_, C-12), 11.2 (CH_3_, C-13); HRESIMS *m*/*z* 251.0666 [M + H]^+^ (calcd. for C_13_H_15_O_5_, 251.0642).

4-(Hydroxymethyl)-3-methoxy-5-methylcyclopent-2-enone (**9**): White amorphous solid; [α]^2^_D_ −4.2 (*c* 0.12, MeOH); UV (MeOH) λ_max_ (log ε) 237 (2.93) nm; IR (KBr) ν_max_: 3389, 2962, 2934, 2877, 1678, 1590, 1515, 1455, 1439, 1383, 1360, 1255, 1195 cm^−1^; ^1^H-NMR (500 MHz, DMSO-*d*_6_) δ_H_: 5.32 (1H, s, H-2), 4.77 (1H, t, *J *= 5.0 Hz, 7-OH), 3.80 (3H, s, OCH_3_), 3.61 (2H, m, CH_2_-7), 2.47 (1H, ddd, *J* = 1.0, 3.0, 6.5 Hz, H-4), 2.32 (1H, qd, *J *= 7.5, 3.0 Hz, H-5), 1.06 (3H, d, *J* = 7.5 Hz, Me-8), ^13^C-NMR (125 MHz, DMSO-*d*_6_) δ_C_: 205.9 (C, C-1), 188.8 (C, C-3), 103.4 (CH, C-2), 59.5 (CH_2_, C-7), 58.7 (CH_3_, C-6), 50.9 (CH, C-5), 42.8 (CH, C-4), 15.3 (CH_3_, C-8); HRESIMS *m*/*z* 157.0854 [M + H]^+^ (calcd. for C_8_H_13_O_3_, 157.0859).

8-Methoxy-1-naphthyl 6′-O-acetyl-β-glucopyranoside (**10**): Yellow amorphous solid; [α]^2^_D_ −16.6 (*c *0.10, MeOH); UV (MeOH) λ_max_ (log ε) 225(3.47), 284(2.69) nm; IR (KBr) ν_max_: 3405, 2933, 1723, 1660, 1641, 1455, 1381, 1268, 1074 cm^−1^; ^1^H-NMR (500 MHz, CDCl_3_) δ_H_: 7.58 (1H, dd, *J* = 2.5, 8.0 Hz, H-5), 7.48 (1H, d, *J* = 8.0 Hz, H-7), 7.41 (1H, dd, *J* = 2.5, 8.0 Hz, H-4), 7.38 (1H, br t, *J *= 8.0 Hz, H-6), 7.36 (1H, br t, *J* = 8.0 Hz, H-3), 6.96 (1H, d, *J* = 8.0 Hz, H-2), 4.81 (1H, d, *J *= 7.0 Hz, H-1′), 4.62 (1H, dd, *J* = 4.5, 12.5 Hz, H-6′b), 4.44 (1H, dd, *J* = 1.5, 12.5 Hz, H-6′a), 4.00 (3H, s, Me-11), 3.82 (1H, t, *J* = 9.5, H-3′), 3.72 (1H, m, H-5′), 3.71 (1H, m, H-4′), 3.59 (1H, m, H-2′), 2.17 (3H, s, Me-13); ^13^C-NMR (125 MHz, CDCl_3_) δ_C_: 171.6 (C, COOCH_3_), 155.6 (C, C-8), 155.0 (C, C-1), 136.2 (C, C-10), 126.5 (CH, C-3), 126.4 (CH, C-6), 124.2 (CH, C-5), 122.1 (CH, C-4), 118.4 (C, C-9), 114.7 (CH, C-7), 108.5 (CH, C-2), 105.1 (CH, C-1′), 75.8 (CH, C-2′), 74.5 (CH, C-5′), 74.0 (CH, C-3′), 69.9 (CH, C-4′), 63.3 (CH_2_, C-6′), 57.2 (CH_3_, C-11), 20.9 (CH_3_, C-13); HRESIMS *m*/*z* 401.1209 [M + Na]^+^ (calcd. for C_19_H_22_NaO_8_, 401.1212).

### 3.5. Enzyme-Inhibitory Activity Assays

Enzyme-inhibitory activity assays were tested according to literatures reported [25,26,27,28].

### 3.6. Larval Settlement Assays

Larval culture and larval settlement assays matched the method reported in reference [29].

## 4. Conclusions

In conclusion, our investigation on the chemical constituents of the extract from gorgonian-derived fungal strain* Xylariaceae *sp. SCSGAF0086 led to the obtainment of 18 polyketides (**1**–**18**). Among these compounds, **1**, **2**, **8**, **9**, and **10** were new **4**, **6**, **7**, and **17** showed enzyme-inhibitory activities towards several tested enzymes, and **6** and **7** showed strong antifouling activity against *B. neritina *larvae settlement. This is the first time that the antifouling and enzyme-inhibitory activities of these compounds has been reported. 

## Supplementary Files

Supplementary File 1 Supporting Information (PDF, 5483 KB)
