# Supplementary material for: Polyketides from a Marine-Derived Fungus Xylariaceae sp
Source: Mar Drugs. 2013 May 21;11(5):1718–27. doi: 10.3390/md11051718 (PMC3707170; doi:10.3390/md11051718)
Supplement: Supplementary File 1 — Supporting Information (PDF, 5483 KB) [file marinedrugs-11-01718-s001.pdf]

Supporting Information

Figure S1. ^{1}H NMR spectrum of **1** 2
Figure S2. ^{13}C NMR spectrum of **1** 3
Figure S3. DEPT135 spectrum of **1** 3
Figure S4. HSQC spectrum of **1** 4
Figure S5. HMBC spectrum of **1** 4
Figure S6. NOESY spectrum of **1** 5
Figure S7. HRESIMS spectrum of **1** 5
Figure S8. IR spectrum of **1** 6
Figure S9. ^{1}H NMR spectrum of a mixture of **3** and **2** 6
Figure S10. ^{13}C NMR spectrum of a mixture of **3** and **2** 7
Figure S11. DEPT135 spectrum of a mixture of **3** and **2** 7
Figure S12. HSQC spectrum of a mixture of **3** and **2** 8
Figure S13. HMBC spectrum of a mixture of **3** and **2** 8
Figure S14. NOESY spectrum of **2** 9
Figure S15. HR-ESIMS spectrum of 7-carboxypenicitrinol C (**2**) 9
Figure S16. IR spectrum of 7-carboxypenicitrinol C (**2**) 10
Figure S17. ^{1}H NMR spectrum of **8** 10
Figure S18. ^{13}C NMR spectrum of **8** 11
Figure S19. DEPT135 spectrum of **8** 11
Figure S20. HMBC spectrum of **8** 12
Figure S21. HRESIMS spectrum of **8** 12
Figure S22. IR spectrum of **8** 13
Figure S23. ^{13}CNMR, DEPT135 spectra of **9** 13
Figure S24. HMBC spectrum of **9** 14
Figure S25. NOESY spectrum of **9** 14
Figure S26. HRESIMS spectrum of **9** 15
Figure S27. IR spectrum of **9** 15
Figure S28. ^{1}H NMR spectra of **10** and **11** 16
Figure S29. ^{13}C NMR, DEPT135 spectra of **10** and **11** 17
Figure S30. HMBC spectrum of **10** 18
Figure S31. HRESIMS spectrum of **10** 18
Figure S32. IR spectrum of **10** 19
S1. Enzyme inhibitory assay 19
S2. Larval Settlement Bioassays 20

Figure S1. ^{1}H-NMR spectrum of penicitrinol F (**1**).

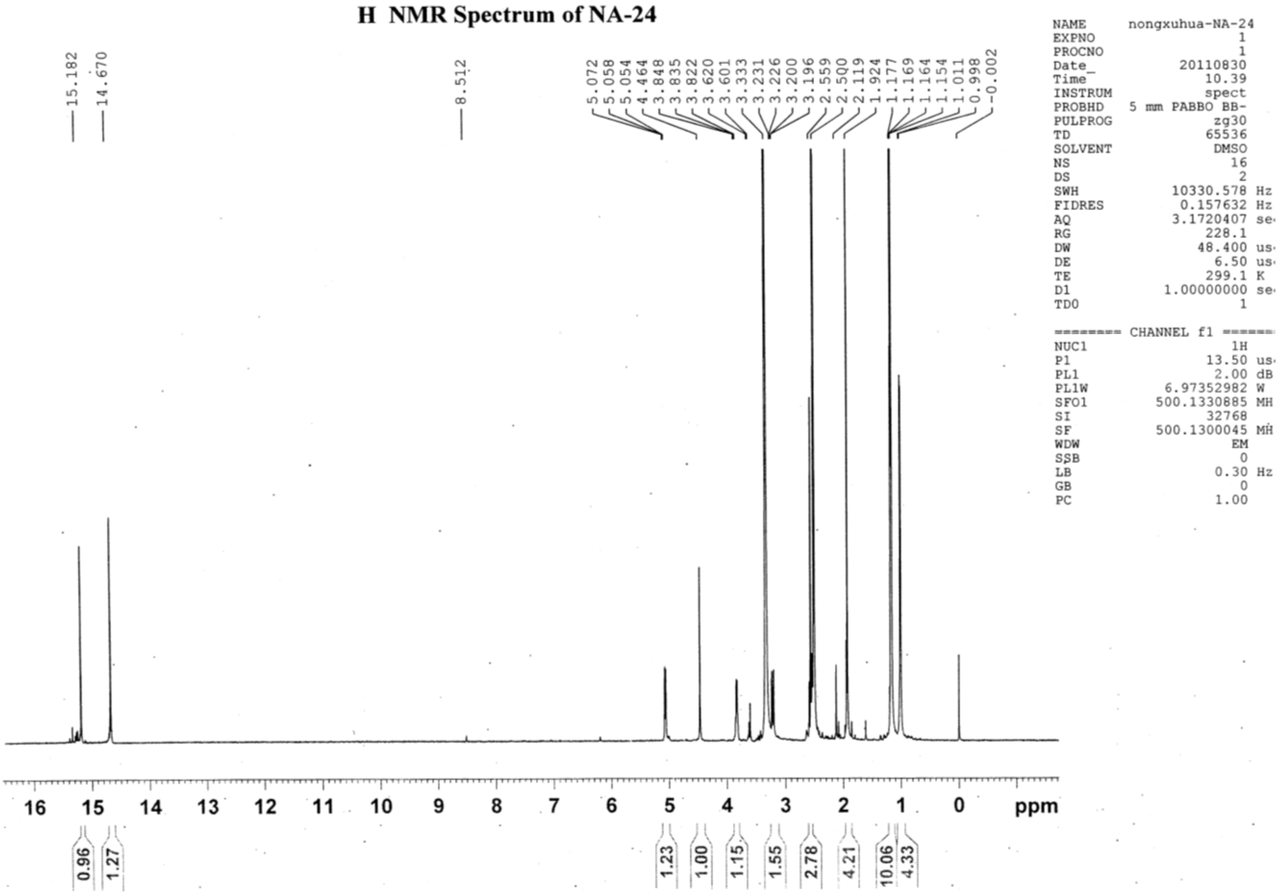

Figure S2. ^{13}C-NMR spectrum of penicitrinol F (**1**).

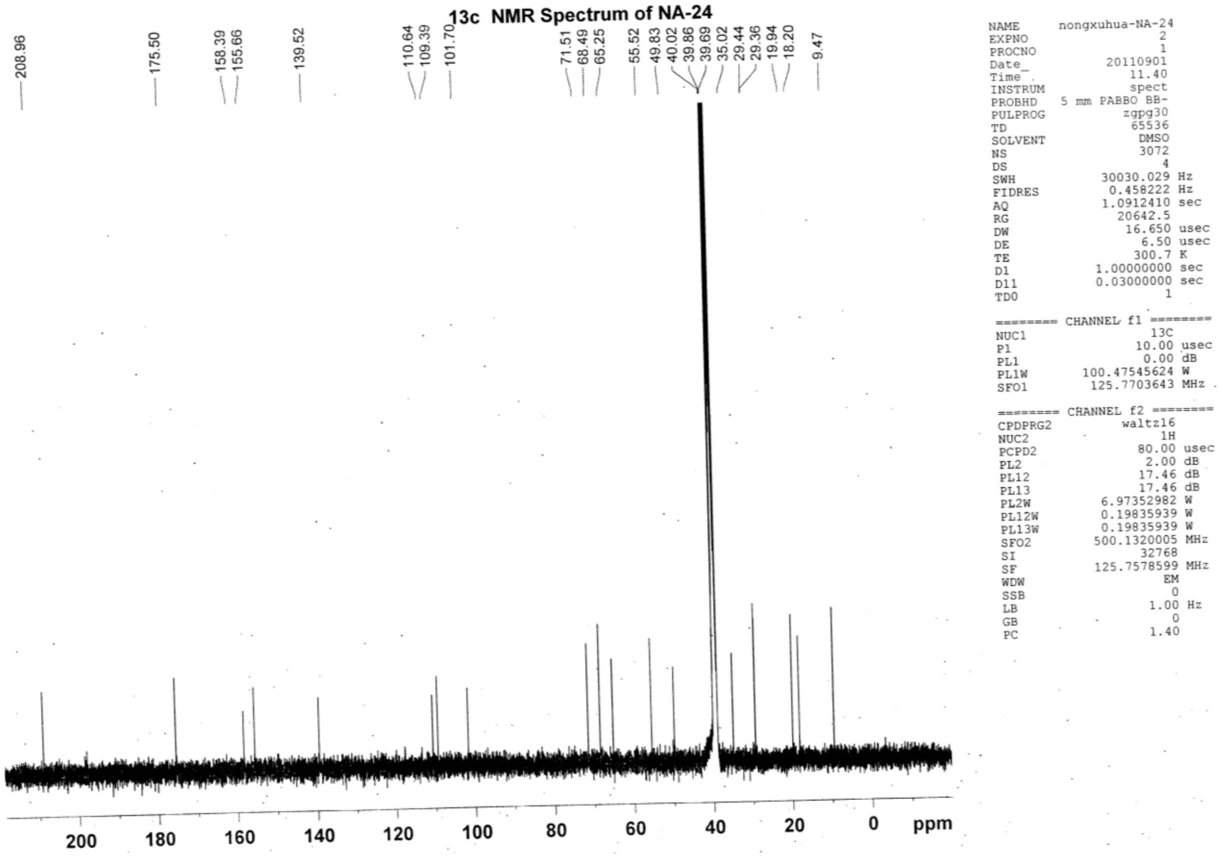

Figure S3. DEPT135 spectrum of penicitrinol F (**1**).

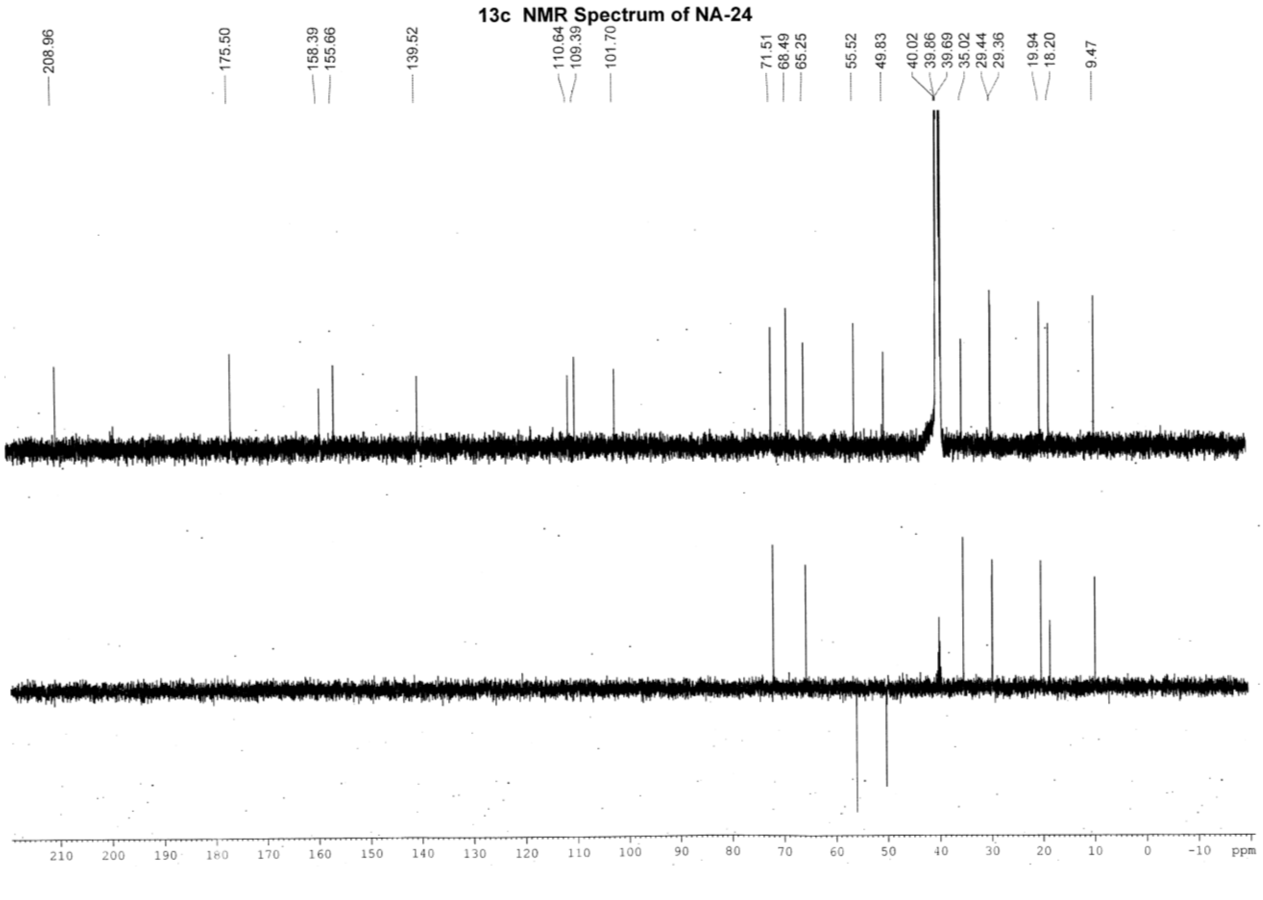

Figure S4. HSQC spectrum of penicitrinol F (**1**).

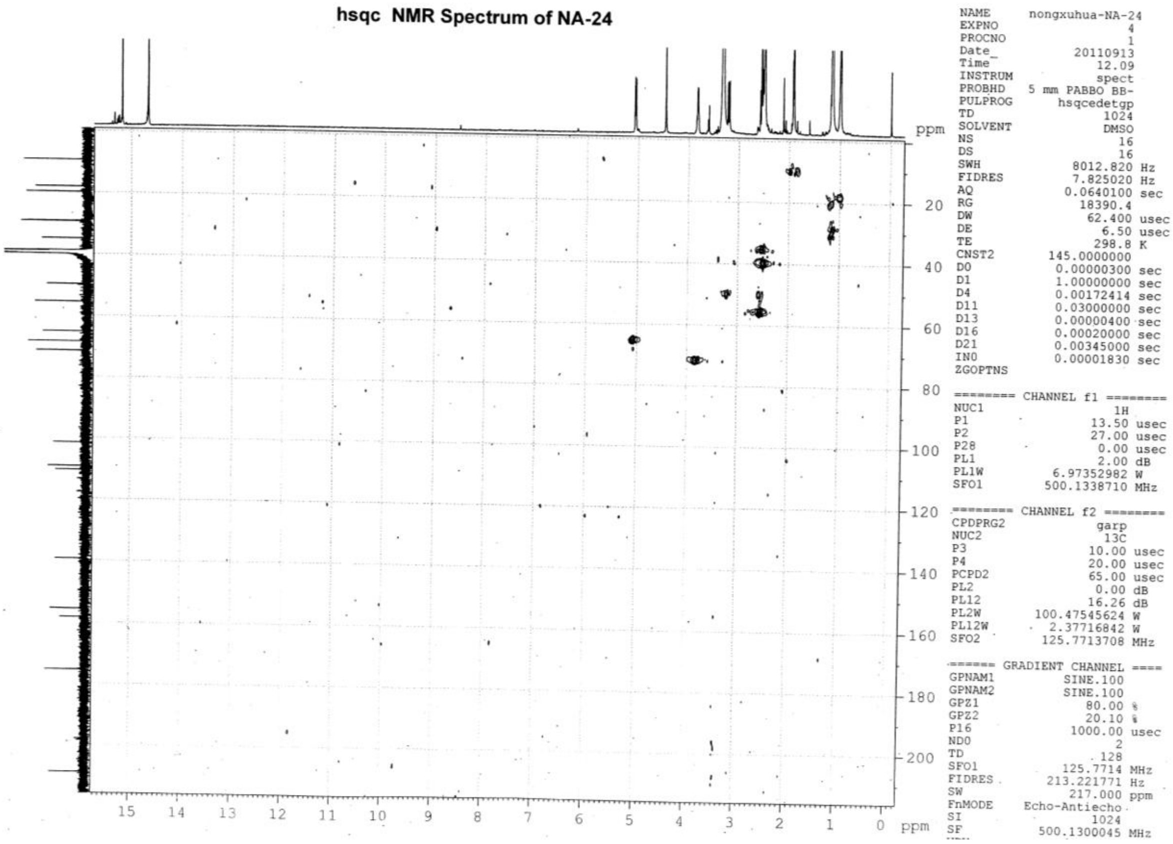

Figure S5. HMBC spectrum of penicitrinol F (**1**).

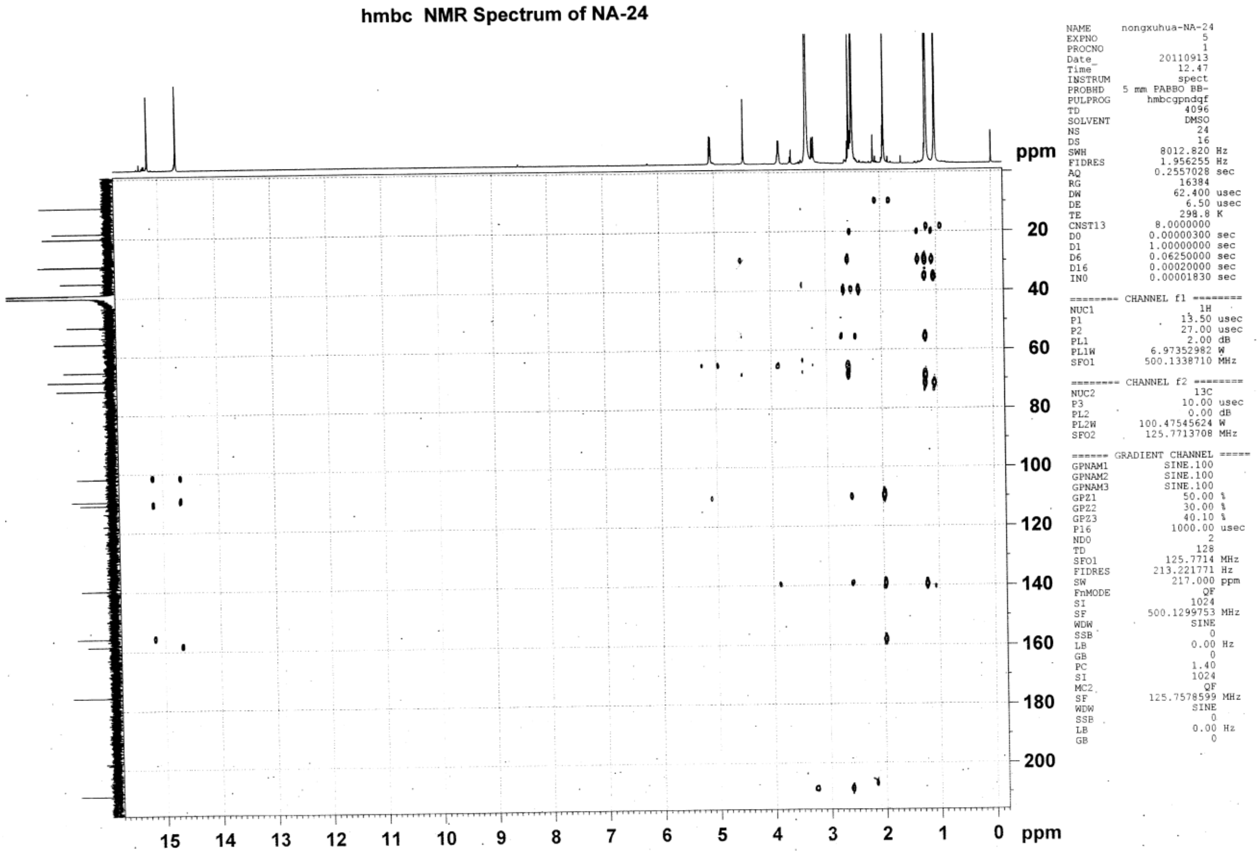

Figure S6. NOE spectrum of penicitrinol F (**1**).

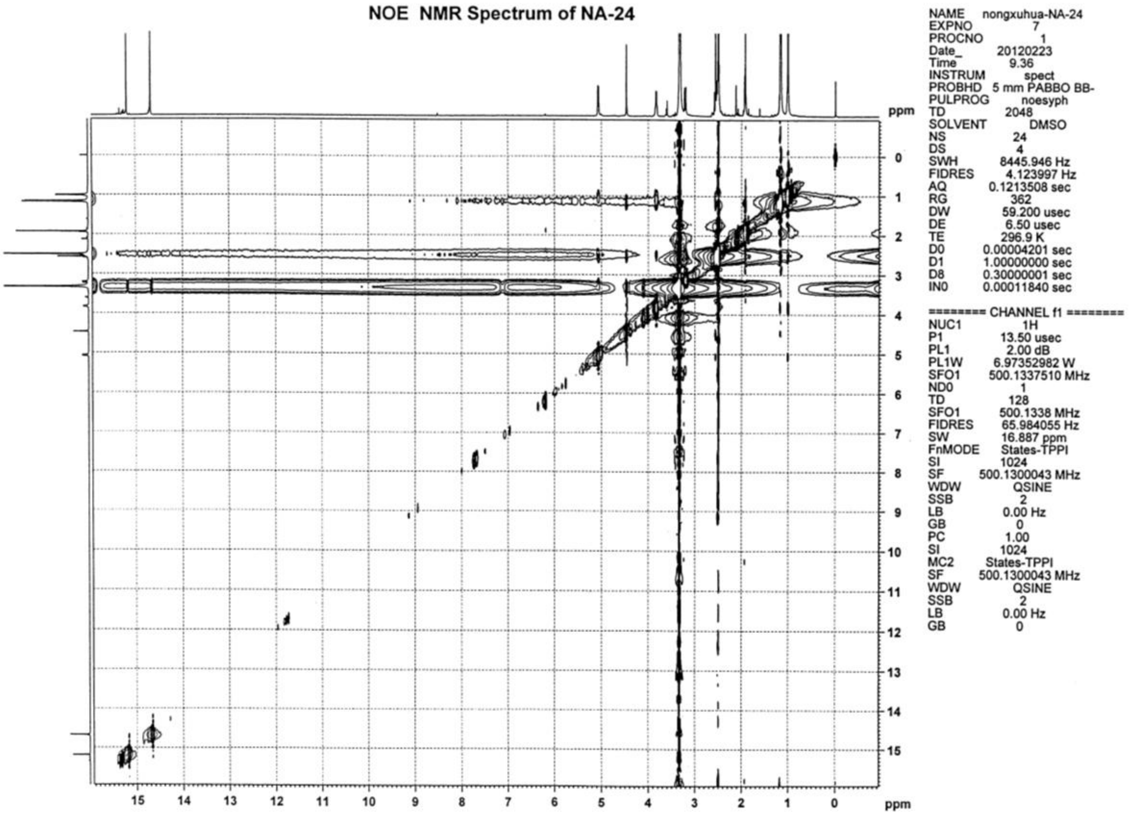

Figure S7. HR-ESIMS spectrum of penicitrinol F (**1**).

Mass Spectrum List Report

Analysis Info

Analysis Name	D:\Data\User\2011\nongxuhua-NA-24A.d	Acquisition Date	12/29/2011 4:40:13 PM
Method	tune_low.m	Operator	BDAL@DE
Sample Name		Instrument / Ser#	maXis 29
Comment			

Acquisition Parameter

Source Type	ESI	Ion Polarity	Positive	Set Nebulizer	0.3 Bar
Focus	Not active	Set Capillary	3500 V	Set Dry Heater	180 °C
Scan Begin	50 m/z	Set End Plate Offset	-500 V	Set Dry Gas	4.0 l/min
Scan End	2200 m/z	Set Collision Cell RF	500.0 Vpp	Set Divert Valve	Waste

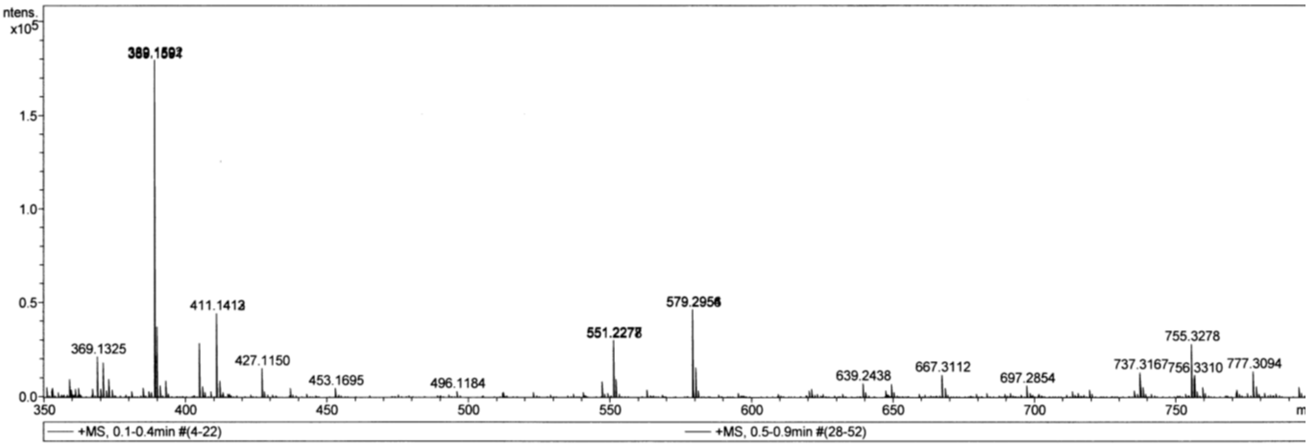

#	m/z	Res.	S/N	I	FWHM
1	230.2489	21819	241.2	8944	0.0106
2	233.0820	26367	404.6	15103	0.0088
3	251.0929	24319	2396.5	94557	0.0103
4	252.0960	23995	313.5	12465	0.0105
5	273.0749	23378	5351.9	220731	0.0117

Bruker Compass DataAnalysis 4.0 printed: 2/27/2012 4:45:32 PM Page 1 of 2

Figure S8. IR spectrum of penicitrinol F (**1**).

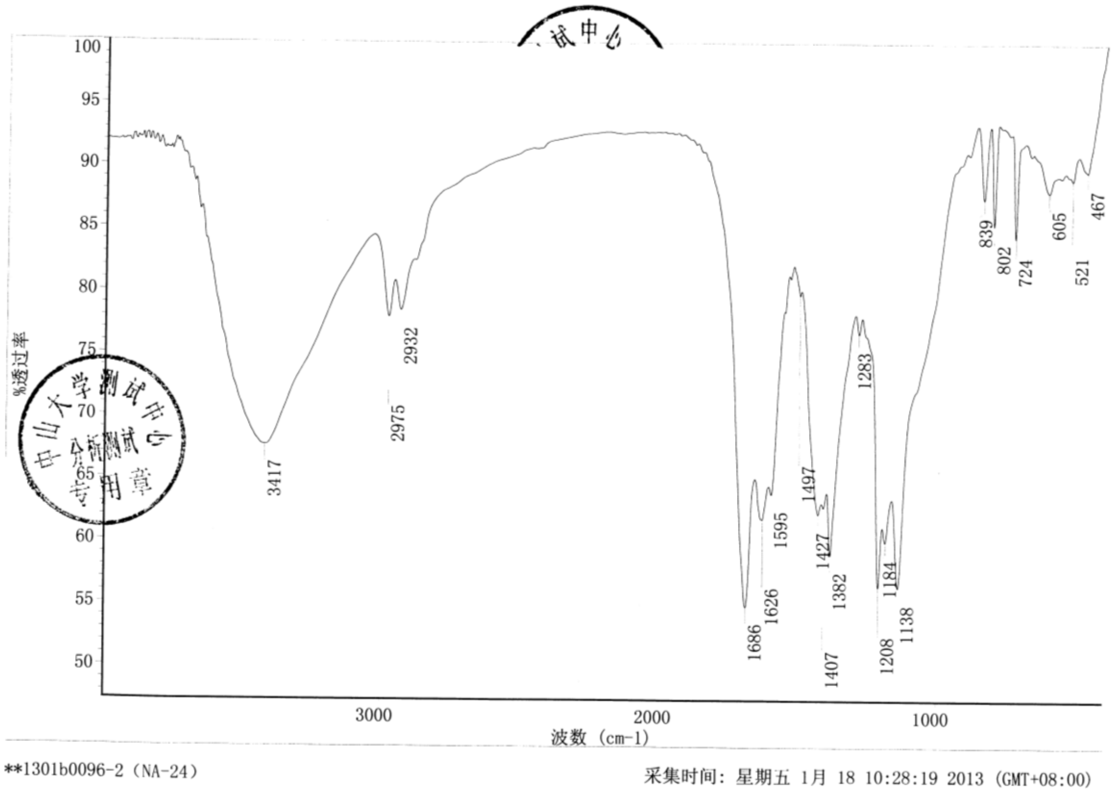

Figure S9. ^{1}H-NMR spectrum of a mixture of **3** and **2**.

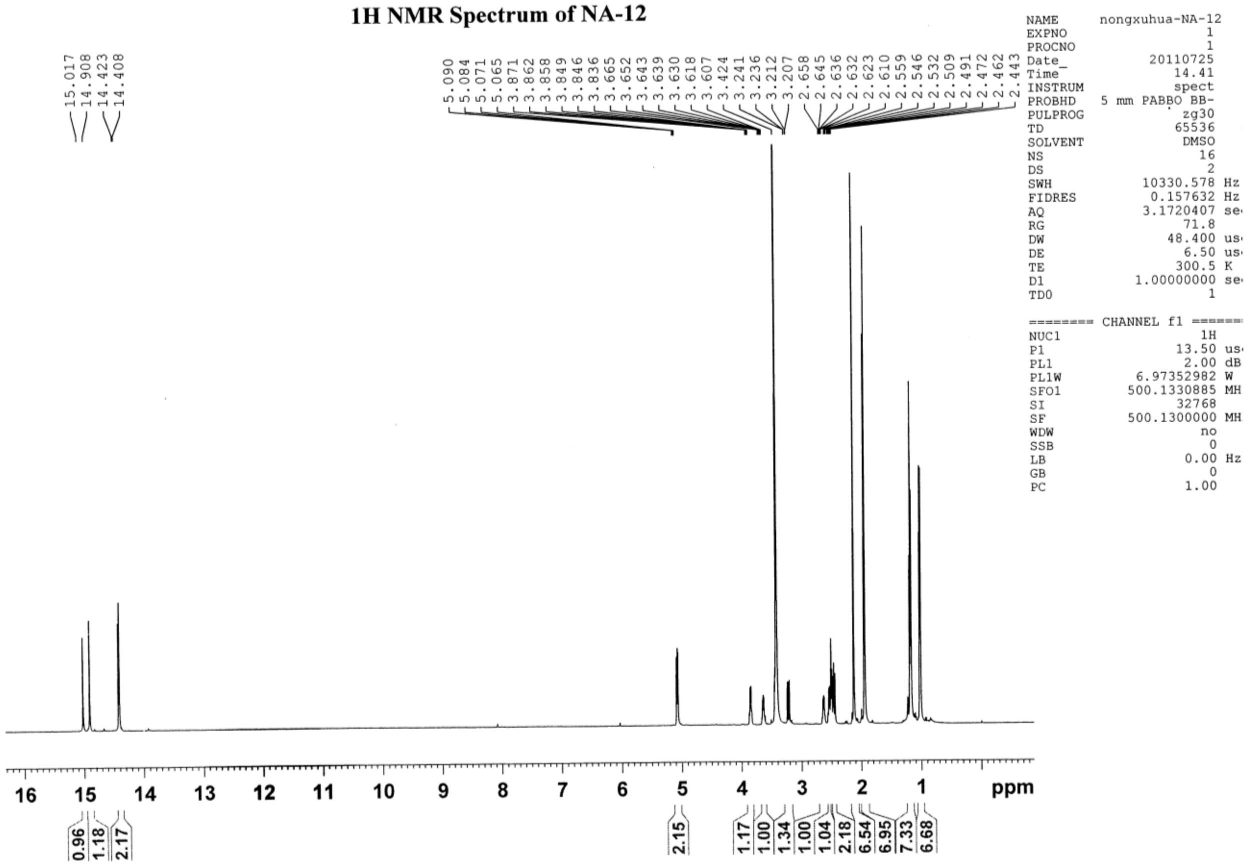

Figure S10. ^{13}C-NMR spectrum of a mixture of **3** and **2**.

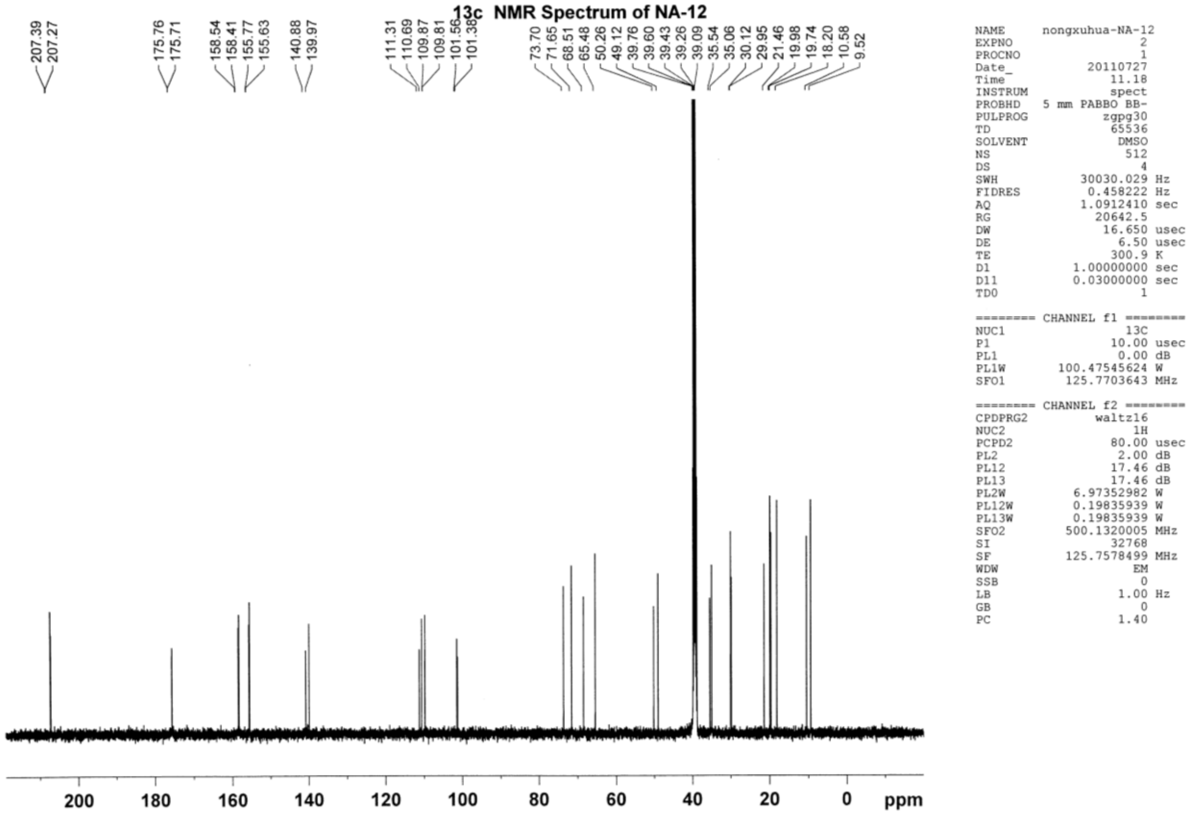

Figure S11. DEPT135 spectrum of a mixture of **3** and **2**.

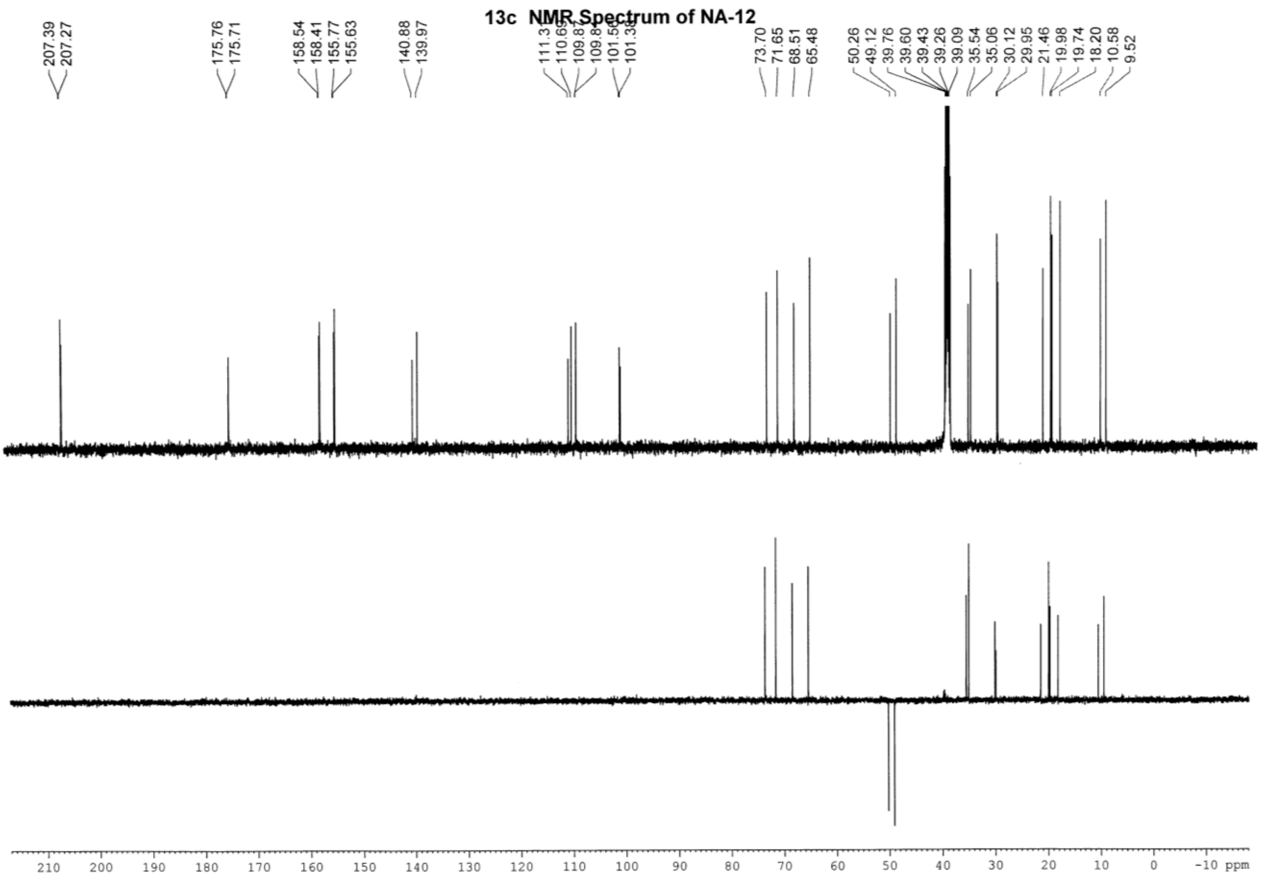

Figure S12. HSQC spectrum of a mixture of **3** and **2**.

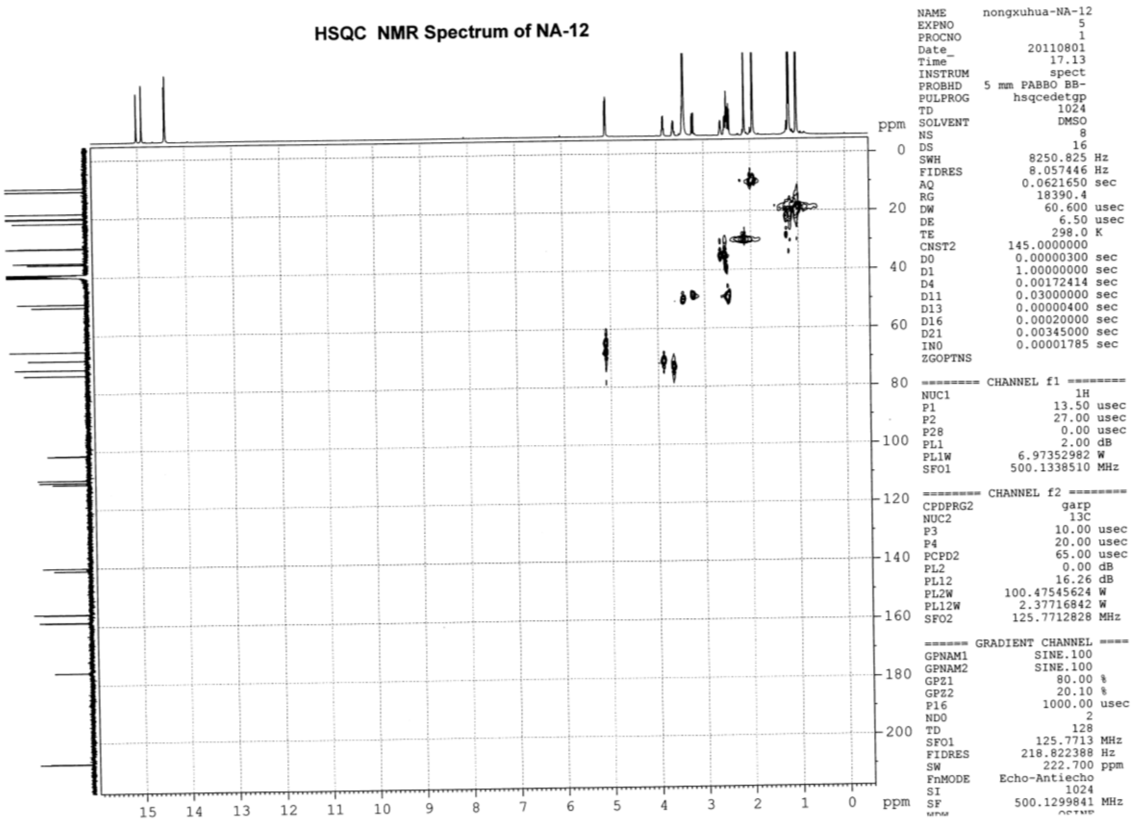

Figure S13. HMBC spectrum of a mixture of **3** and **2**.

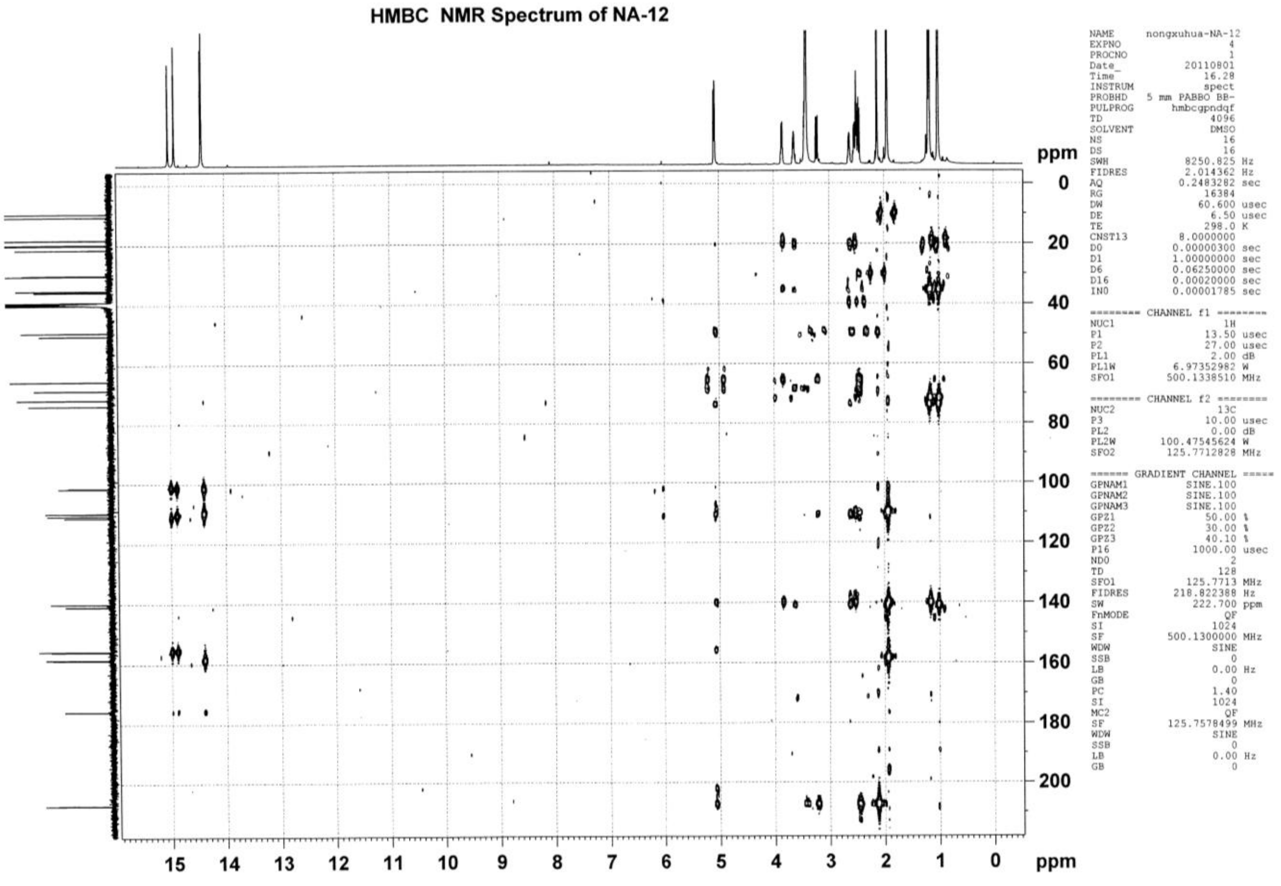

Figure S14. NOE spectrum of 7-carboxypenicitrinol C (**2**).

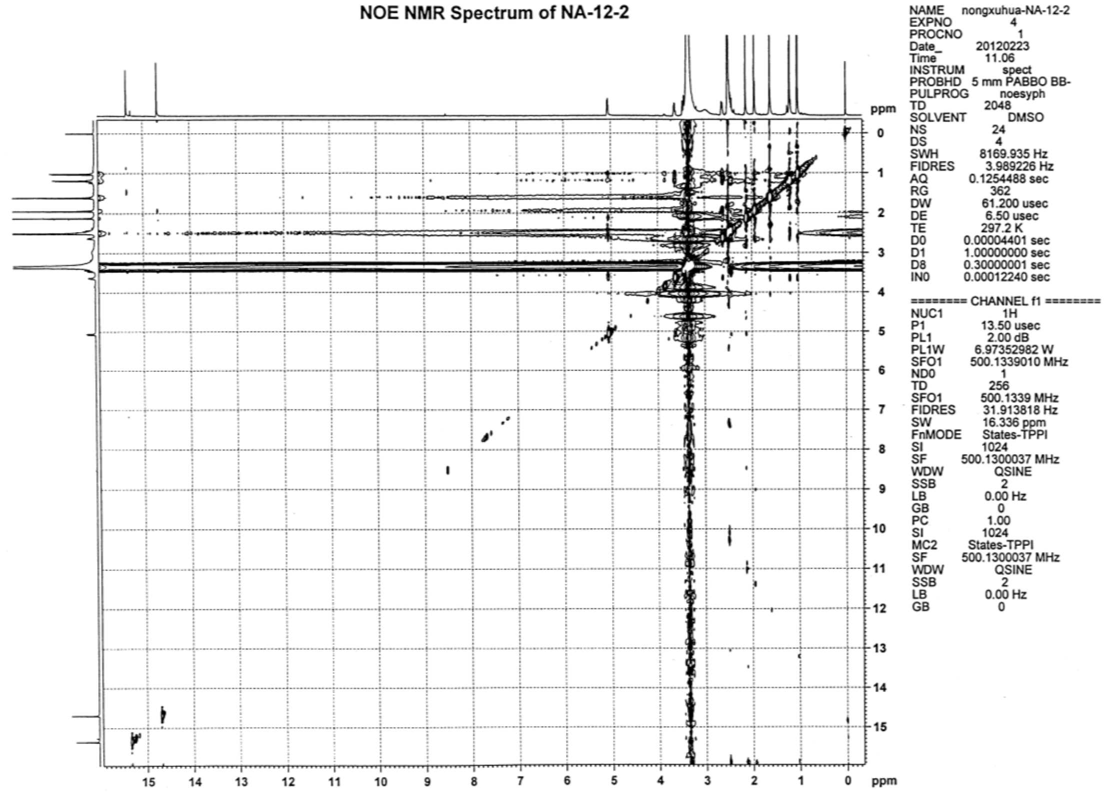

Figure S15. HR-ESIMS spectrum of 7-carboxypenicitrinol C (**2**).

Mass Spectrum List Report

Analysis Info

Analysis Name	D:\Data\User\2011\nongxuhua-NA-12-2.d	Acquisition Date	12/29/2011 5:06:51 PM
Method	tune_low.m	Operator	BDAL@DE
Sample Name		Instrument / Ser#	maXis 29
Comment			

Acquisition Parameter

Source Type	ESI	Ion Polarity	Positive	Set Nebulizer	0.3 Bar
Focus	Not active	Set Capillary	3500 V	Set Dry Heater	180 °C
Scan Begin	50 m/z	Set End Plate Offset	-500 V	Set Dry Gas	4.0 l/min
Scan End	2200 m/z	Set Collision Cell RF	500.0 Vpp	Set Divert Valve	Waste

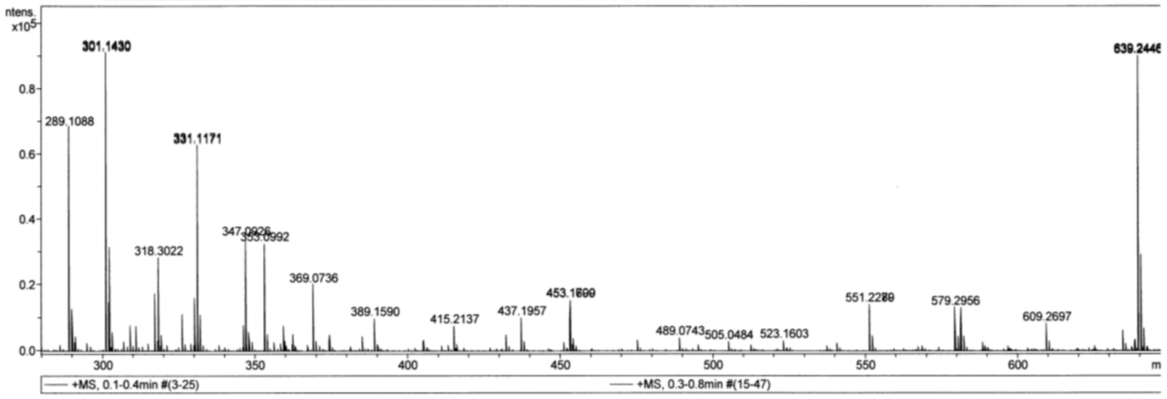

#	m/z	Res.	S/N	I	FWHM
1	149.0242	18876	1059.9	11951	0.0079
2	207.1027	23542	745.8	15501	0.0088
3	233.0823	25485	1481.4	37079	0.0091
4	245.1188	24350	3525.8	95185	0.0101
5	246.1220	22945	496.9	13503	0.0107

Bruker Compass DataAnalysis 4.0 printed: 2/27/2012 4:43:14 PM Page 1 of 2

Figure S16. IR spectrum of 7-carboxypenicitrinol C (**2**).

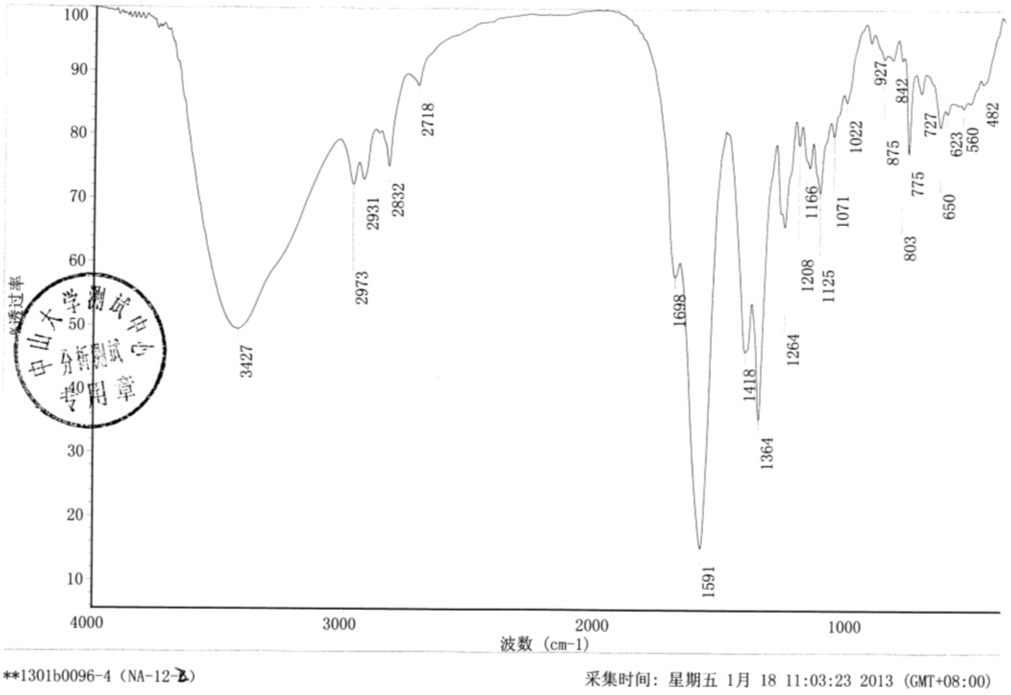

Figure S17. ¹H-NMR spectrum of 2,6-dihydroxy-4,5-dimethyl-3-(3-oxo-1-butenyl) benzoic acid (**8**).

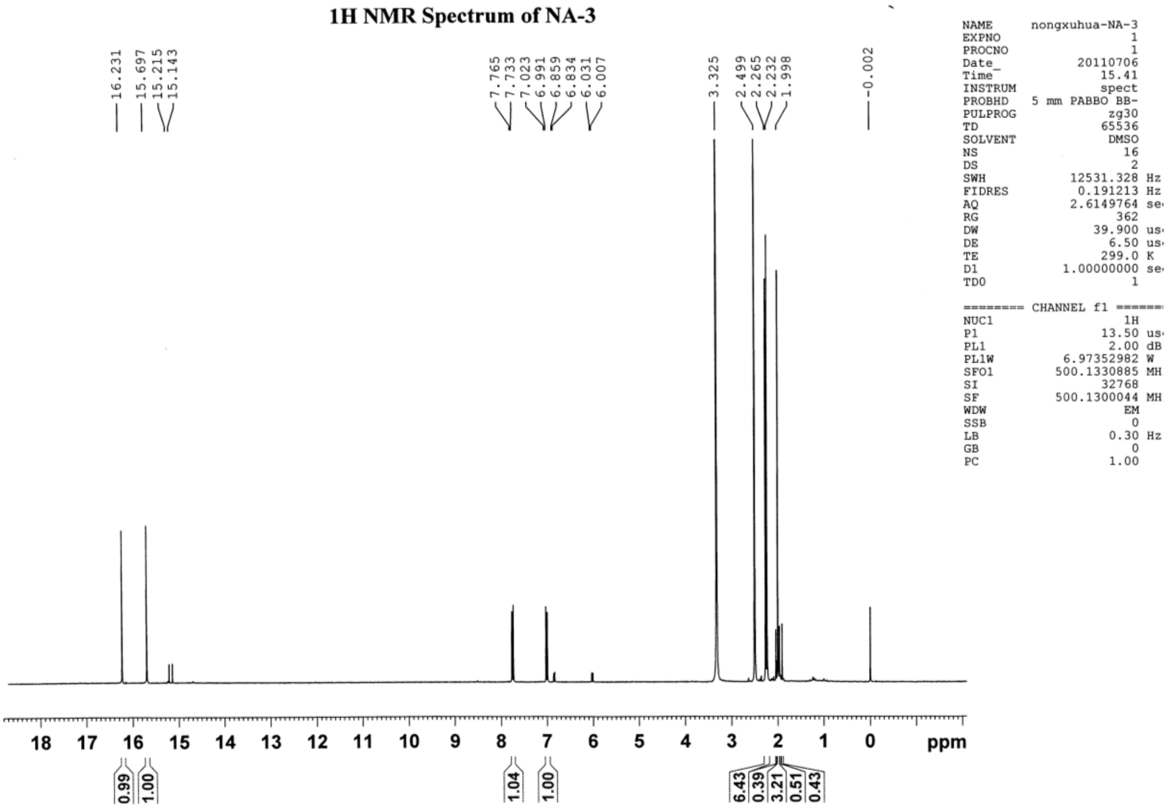

Figure S18. ^{13}C-NMR spectrum of 2,6-dihydroxy-4,5-dimethyl-3-(3-oxo-1-butenyl) benzoic acid (**8**).

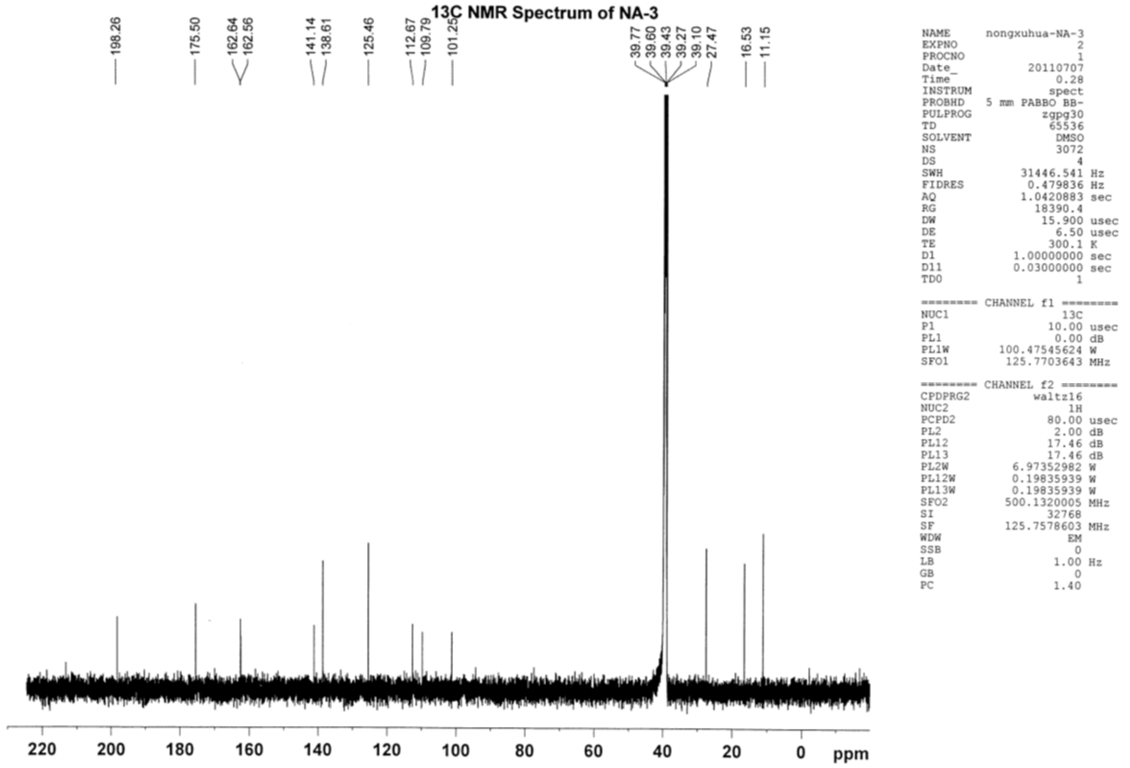

Figure S19. DEPT135 spectrum of 2,6-dihydroxy-4,5-dimethyl-3-(3-oxo-1-butenyl) benzoic acid (**8**).

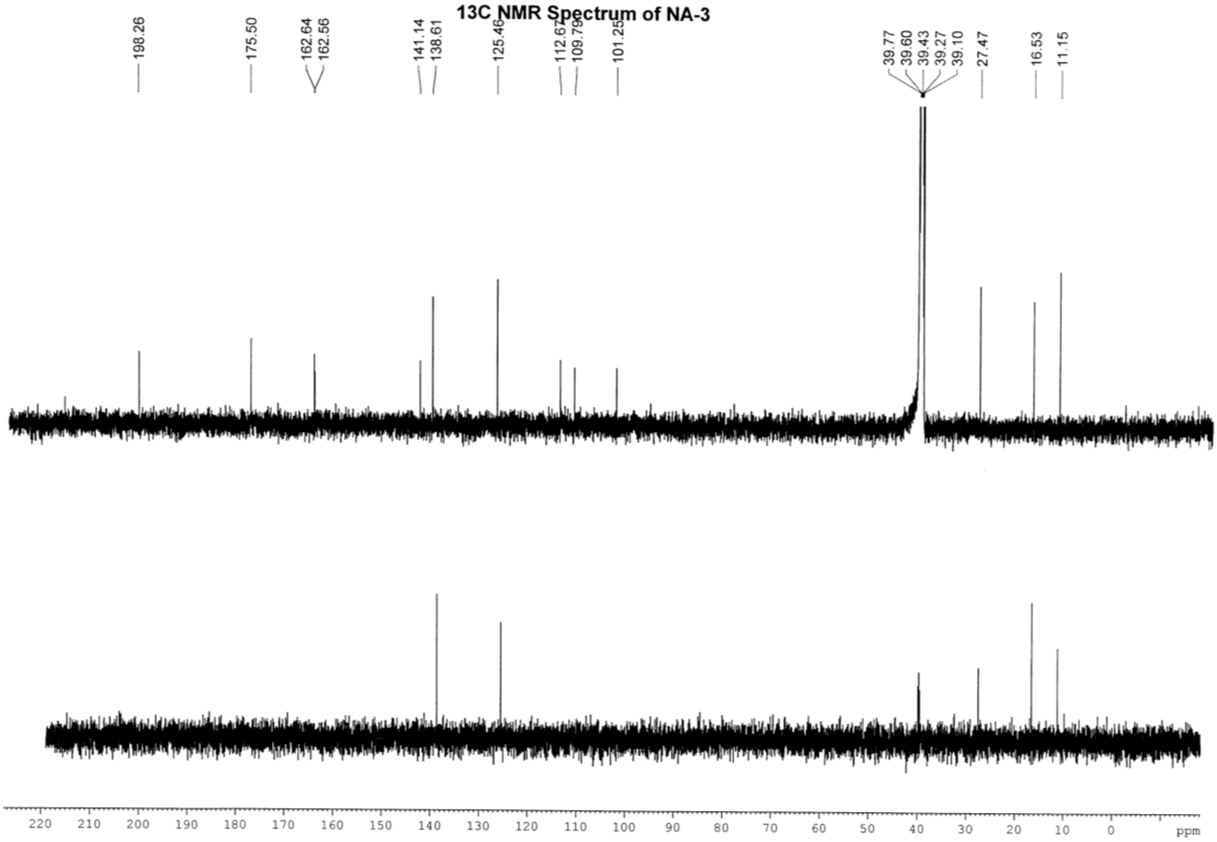

Figure S20. HMBC spectrum of 2,6-dihydroxy-4,5-dimethyl-3-(3-oxo-1-butenyl)benzoic acid (**8**).

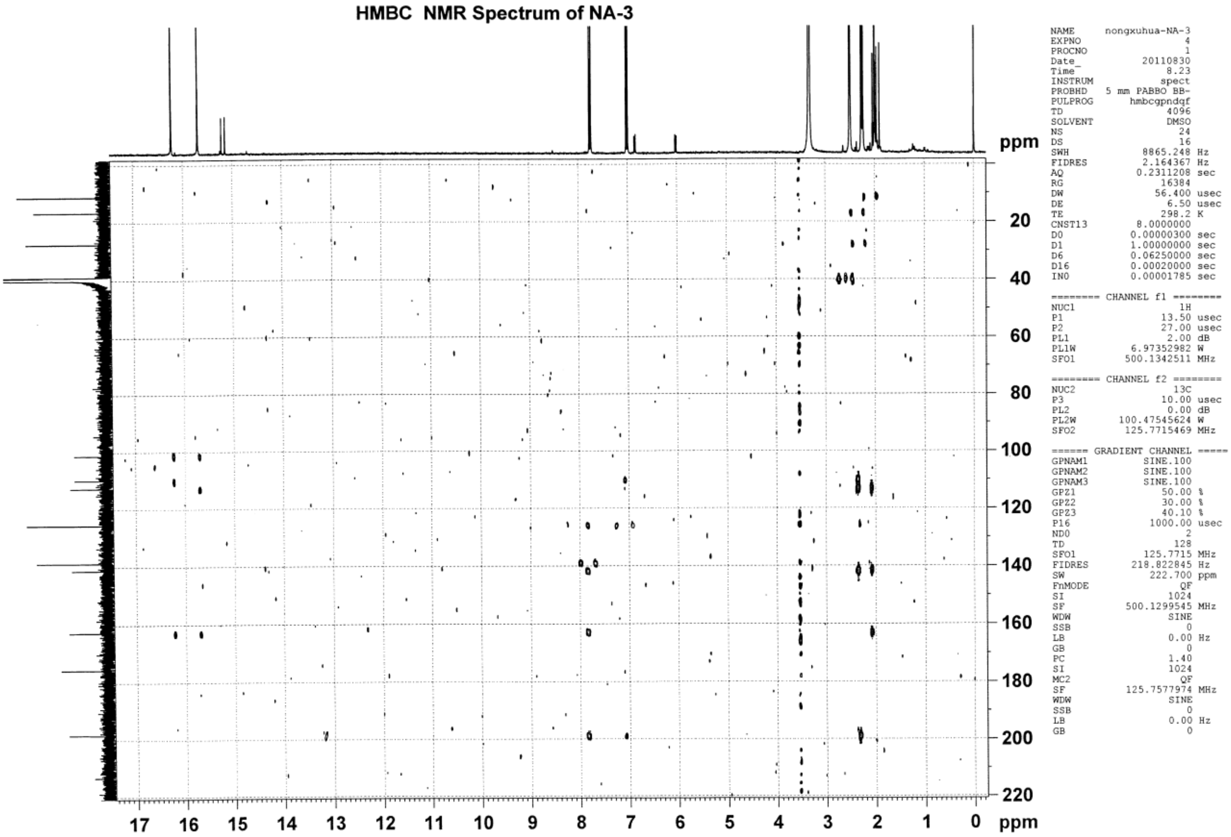

Figure S21. HR-ESIMS spectrum of 2,6-dihydroxy-4,5-dimethyl-3-(3-oxo-1-butenyl) benzoic acid (**8**).

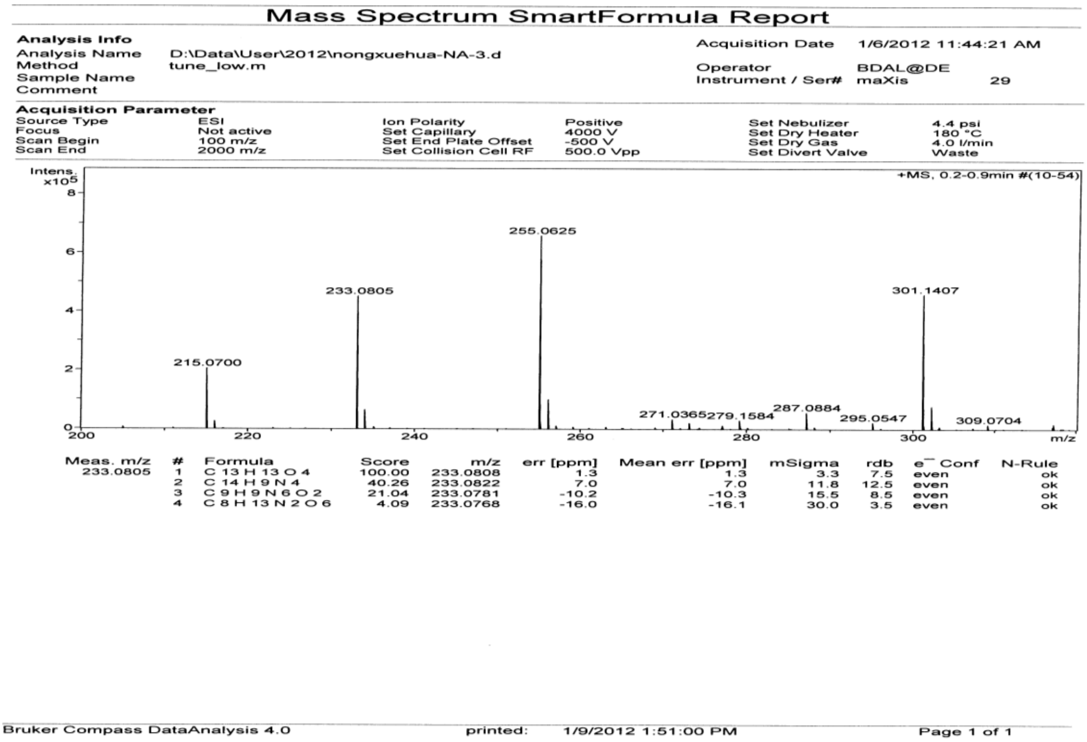

Figure S22. IR spectrum of 2,6-dihydroxy-4,5-dimethyl-3-(3-oxo-1-butenyl)benzoic acid (**8**).

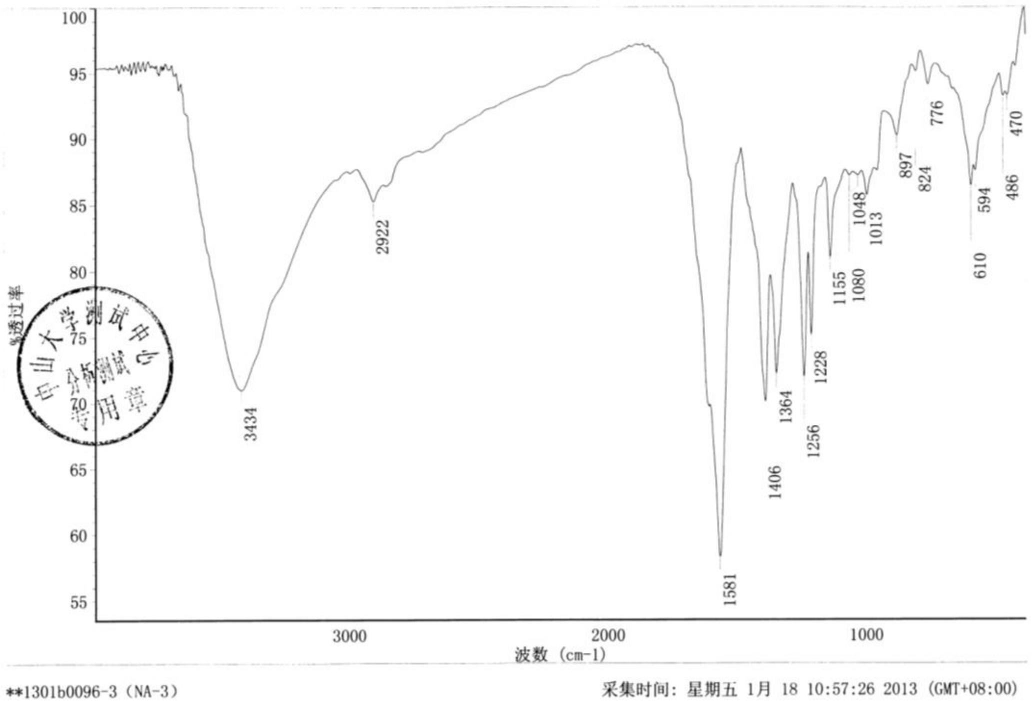

Figure S23. ^{13}C-NMR, DEPT135 spectra of 4-(hydroxymethyl)-3-methoxy-5-methyl cyclopent-2-enone (**9**).

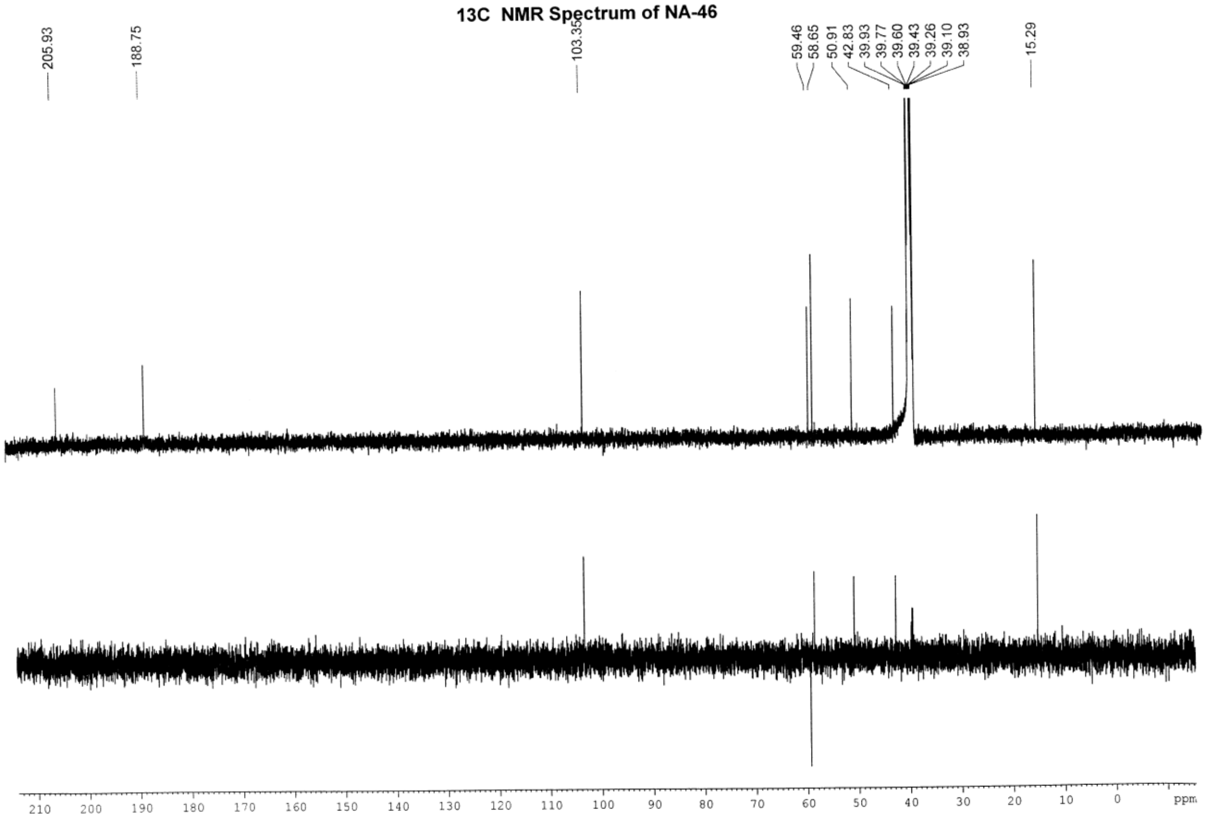

Figure S24. HMBC spectrum of 4-(hydroxymethyl)-3-methoxy-5-methylcyclopent-2-enone (**9**).

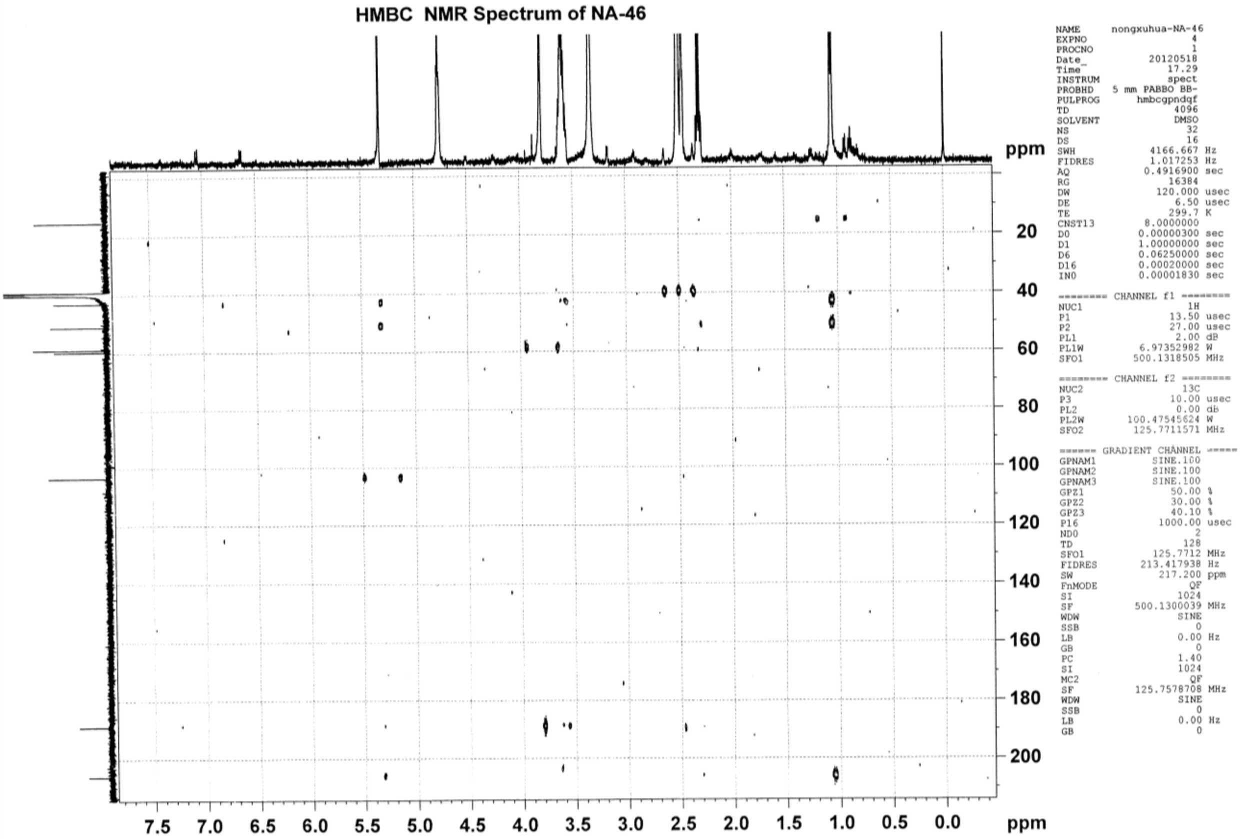

Figure S25. NOE spectrum of 4-(hydroxymethyl)-3-methoxy-5-methylcyclopent-2-enone (**9**).

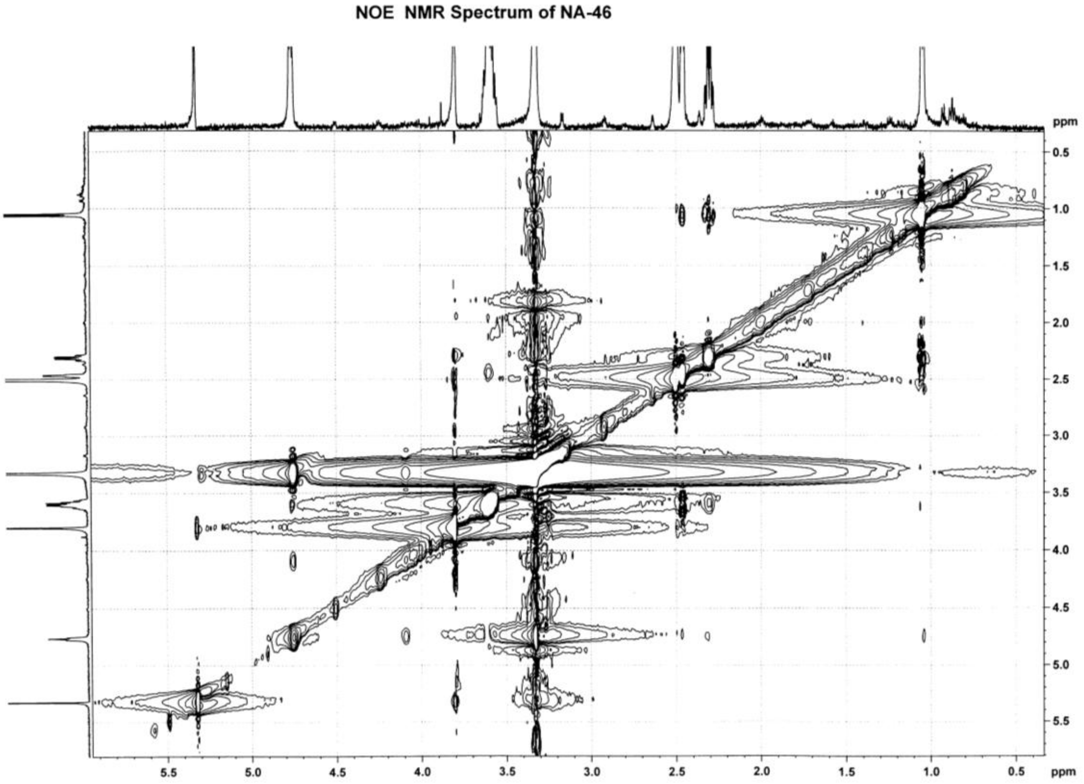

Figure S26. HRESIMS spectrum of 4-(hydroxymethyl)-3-methoxy-5-methyl cyclopent-2-enone (**9**).

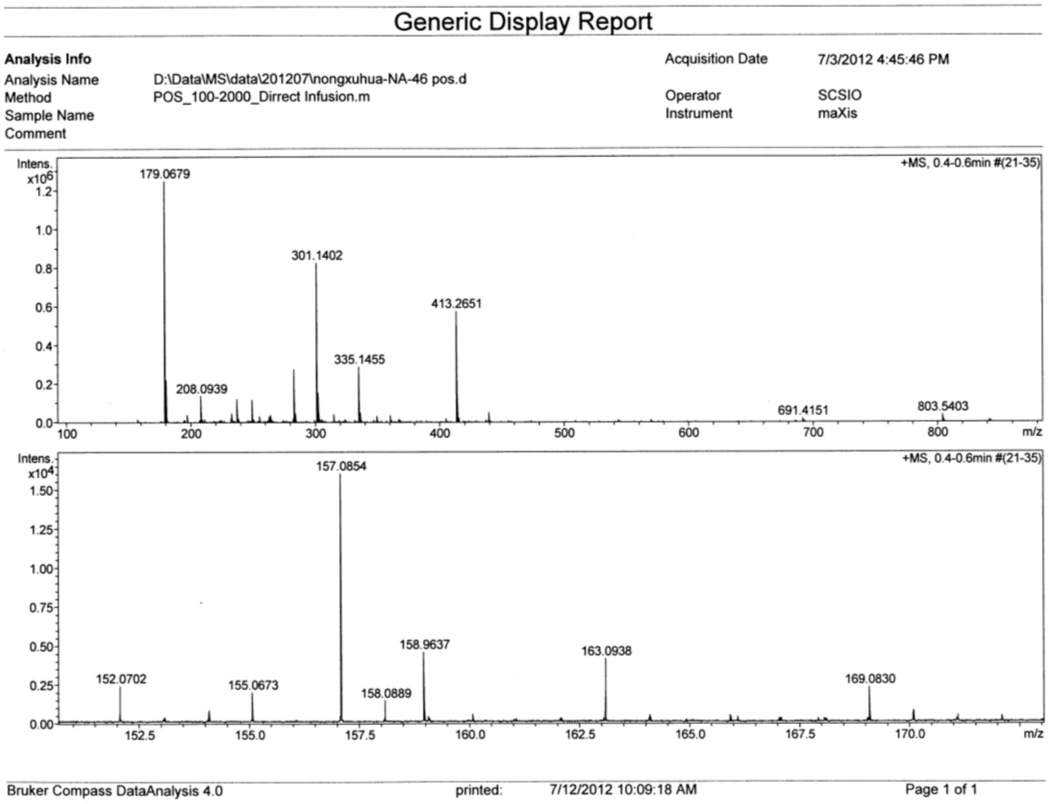

Figure S27. IR spectrum of 4-(hydroxymethyl)-3-methoxy-5-methylcyclopent-2-enone (**9**).

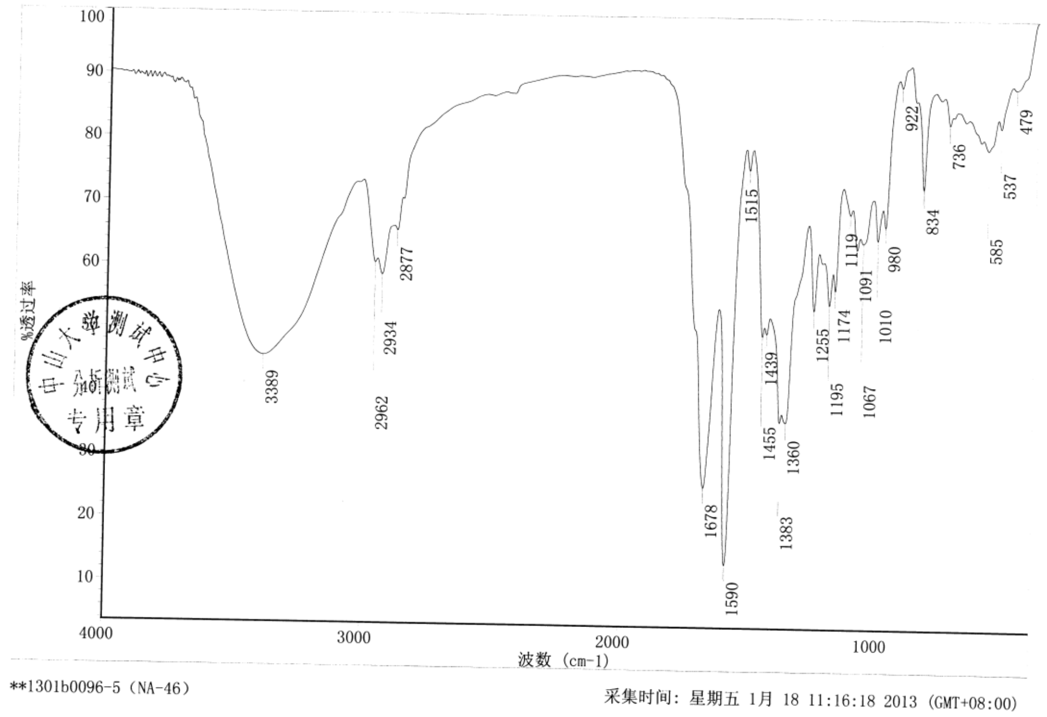

Figure S28. ^{1}H-NMR spectrum of **10** (NA-48) (**A**) and **11** (NA-27) (**B**).

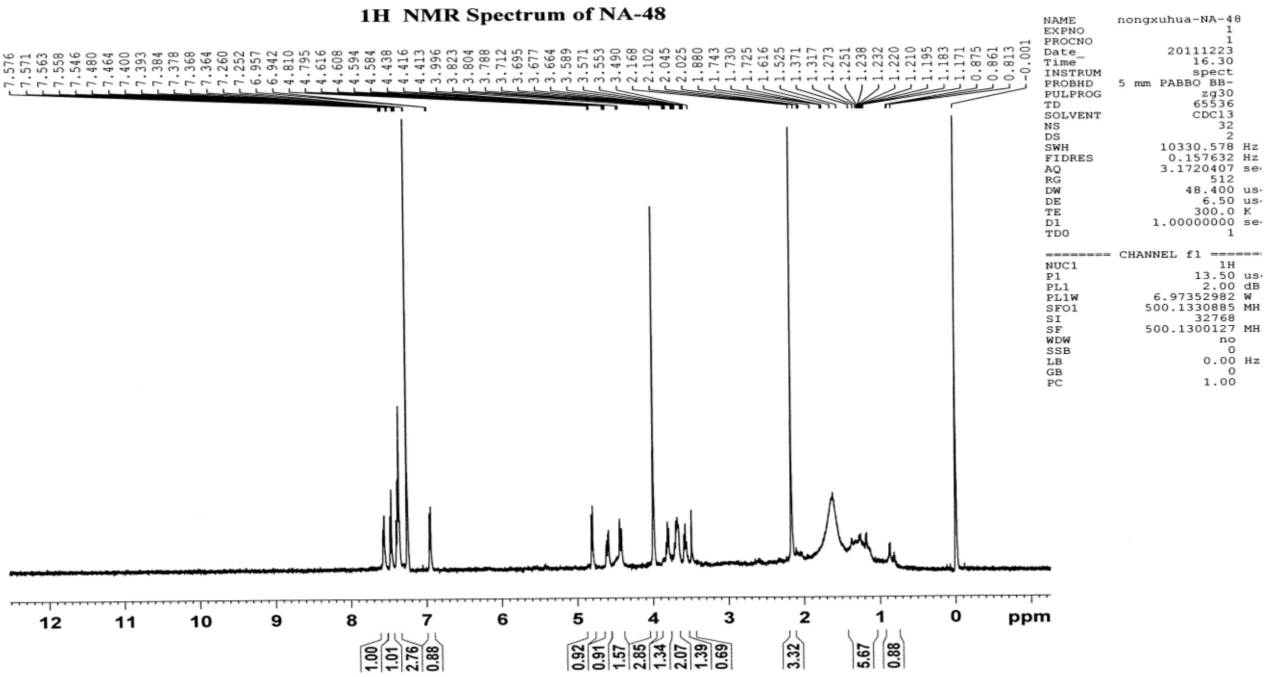

(**A**)

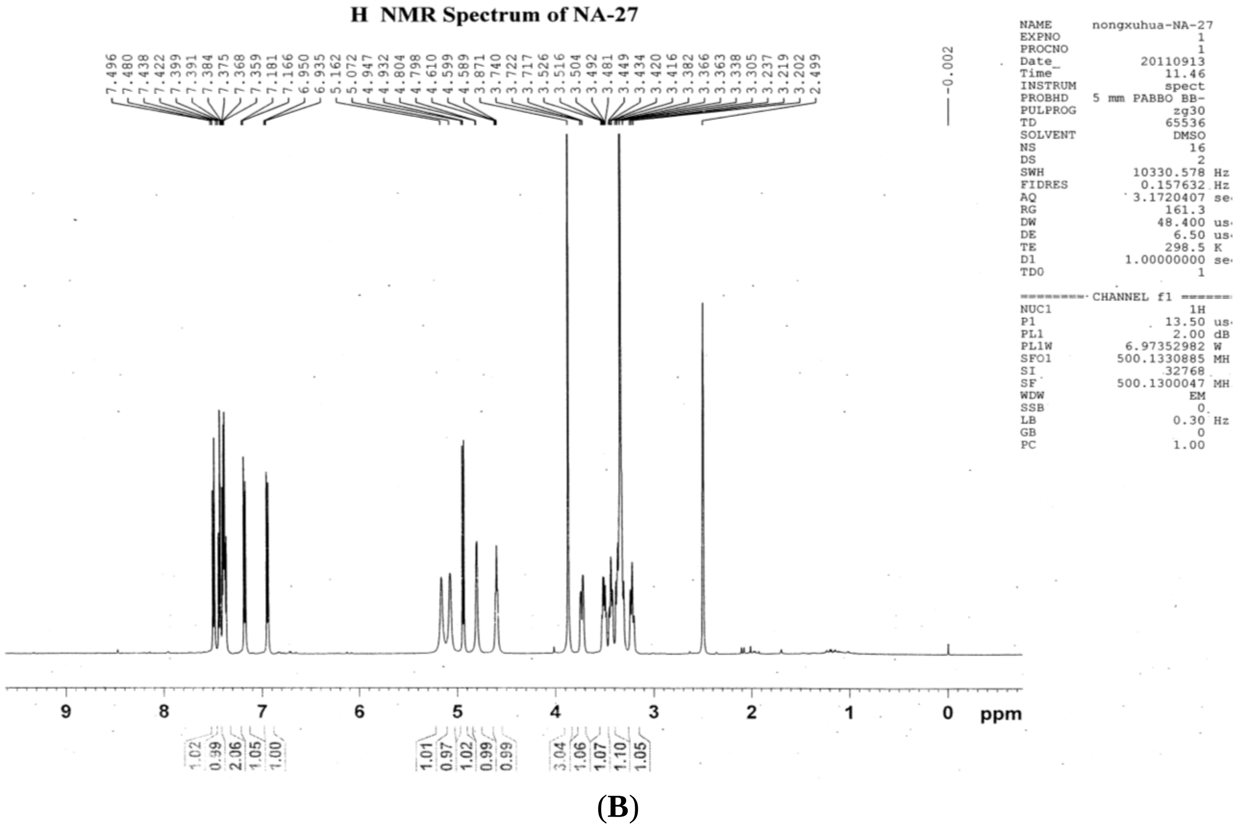

(**B**)

Figure S29. ^{13}C-NMR, DEPT135 spectra of **10** (NA-48) (**A**) and **11** (NA-27) (**B**).

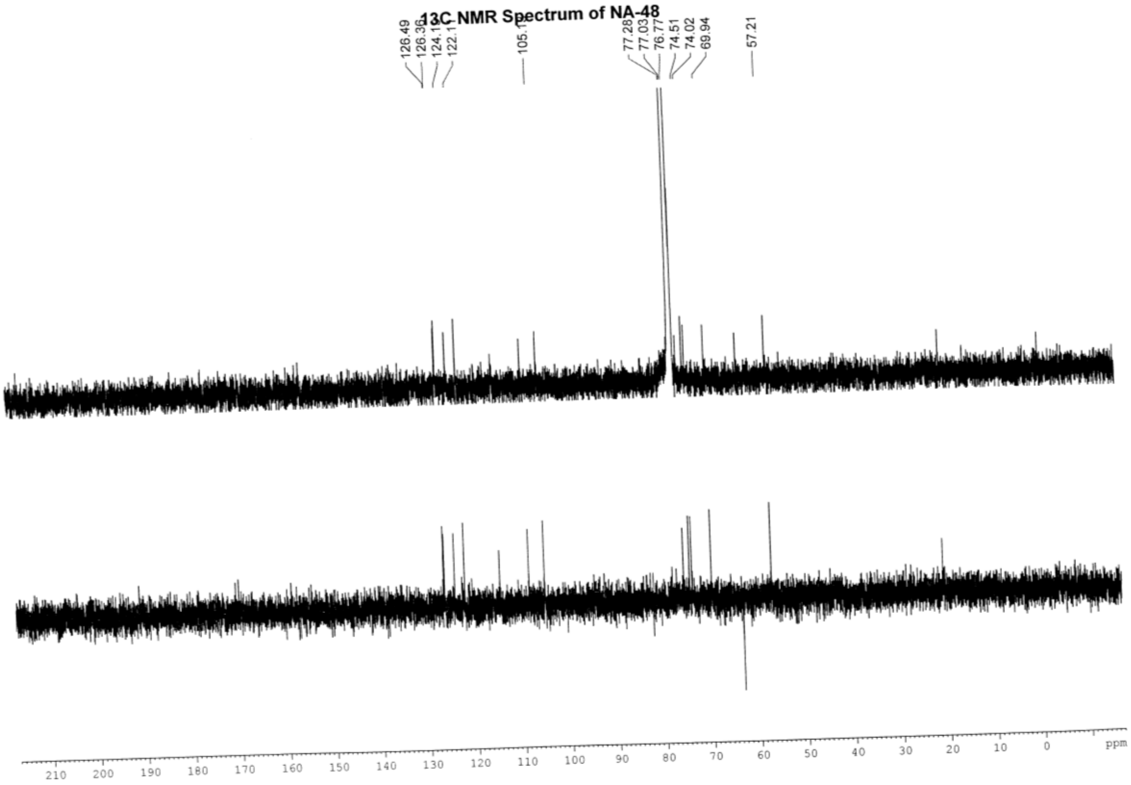

(**A**)

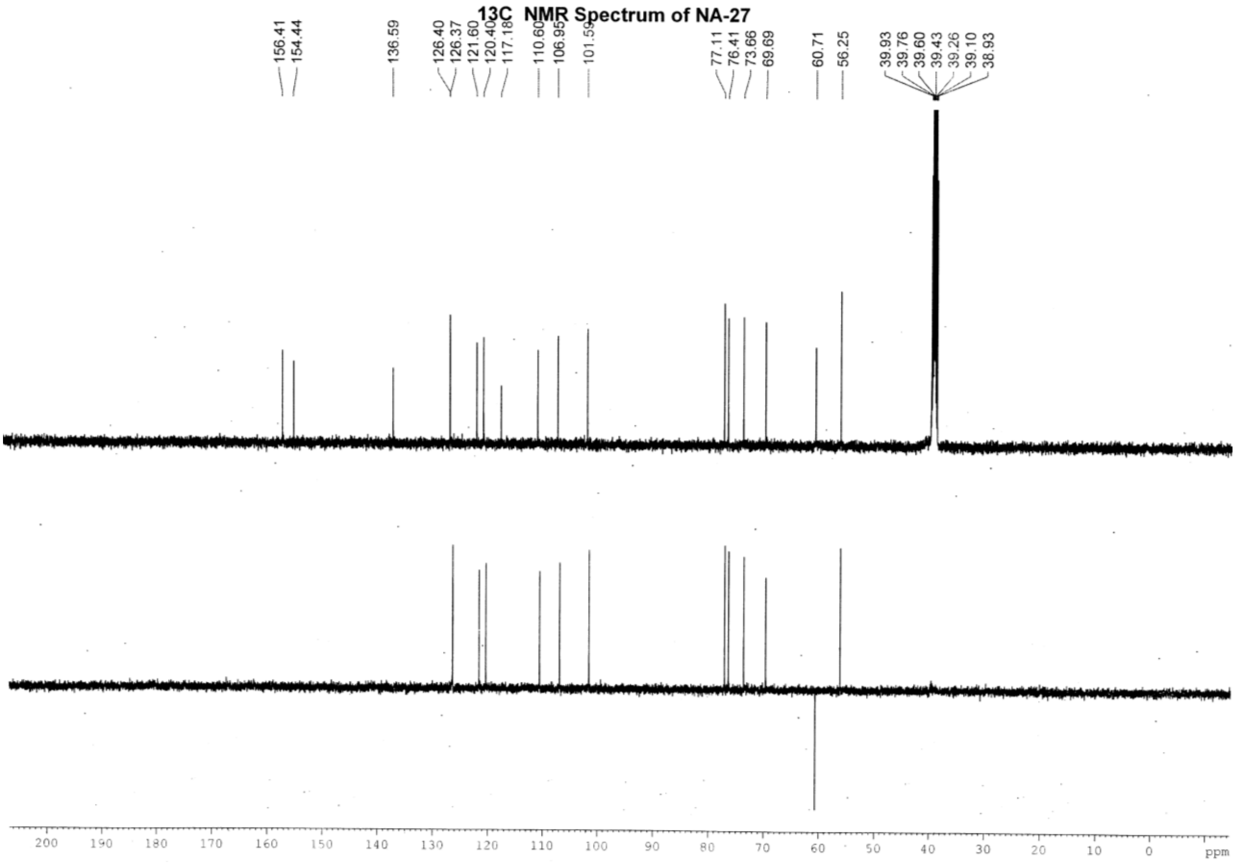

(**B**)

Figure S30. HMBC spectrum of 8-methoxy-1-naphthyl-1-(6′-*O*-acetyl)-α-glucopyranoside (**10**).

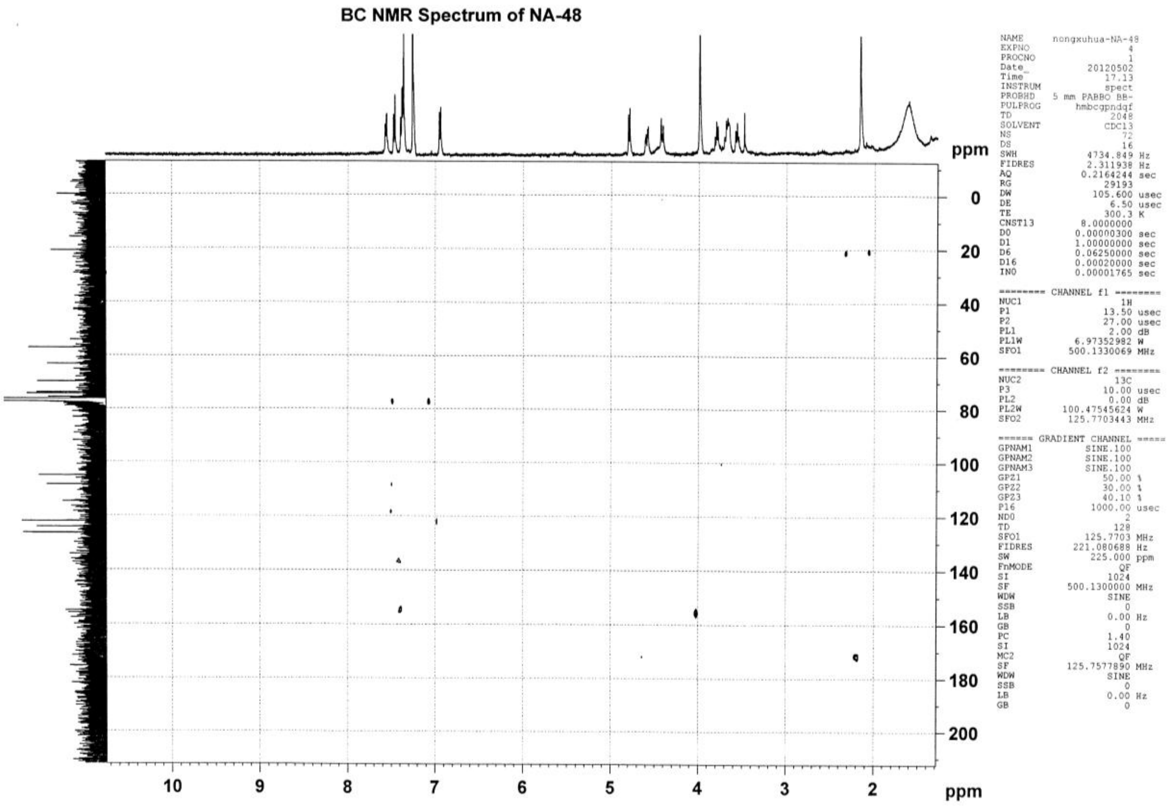

Figure S31. HRESIMS spectrum of 8-methoxy-1-naphthyl-1-(6′-*O*-acetyl)-α-glucopyranoside (**10**).

Mass Spectrum SmartFormula Report

Analysis Info

Analysis Name	D:\Data\MS\data\201207\nongxuhua-NA-48- pos.d	Acquisition Date	7/3/2012 4:32:06 PM
Method	POS_100-2000_Dirrect Infusion.m	Operator	SCSIO
Sample Name		Instrument / Ser#	maXis 29
Comment			

Acquisition Parameter

Source Type	ESI	Ion Polarity	Positive	Set Nebulizer	0.3 Bar
Focus	Not active	Set Capillary	3500 V	Set Dry Heater	180 °C
Scan Begin	50 m/z	Set End Plate Offset	-500 V	Set Dry Gas	4.0 l/min
Scan End	1200 m/z	Set Collision Cell RF	2000.0 Vpp	Set Divert Valve	Waste

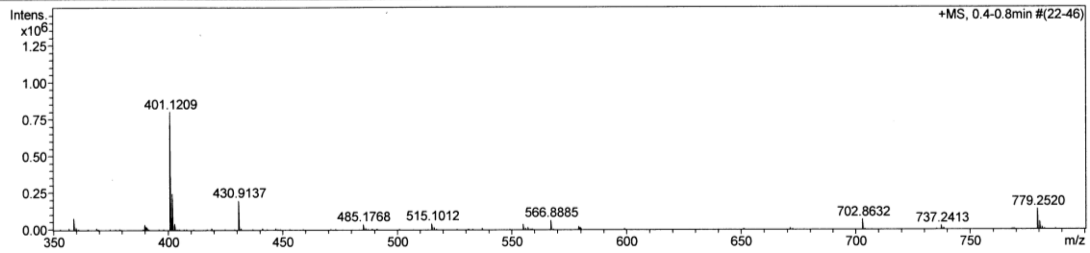

Meas. m/z	#	Formula	Score	m/z	err [mDa]	err [ppm]	mSigma	rdb	e⁻ Conf	N-Rule
401.1209	1	C 20 H 18 N 4 Na O 4	98.21	401.1220	1.1	2.8	46.0	13.5	even	ok
	2	C 16 H 14 N 10 Na O 2	49.05	401.1193	-1.6	-3.9	59.1	14.5	even	ok
	3	C 19 H 22 Na O 8	100.00	401.1207	-0.2	-0.5	60.1	8.5	even	ok
779.2520	1	C 38 H 44 Na O 16	100.00	779.2522	0.2	0.3	7.0	16.5	even	ok
	2	C 39 H 40 N 4 Na O 12	38.71	779.2535	1.5	2.0	18.2	21.5	even	ok
	3	C 40 H 36 N 8 Na O 8	10.49	779.2548	2.9	3.7	28.4	26.5	even	ok

Bruker Compass DataAnalysis 4.0 printed: 7/3/2012 4:41:06 PM Page 1 of 1

Figure S32. IR spectrum of 8-methoxy-1-naphthyl-1-(6′-*O*-acetyl)-α-glucopyranoside (**10**).

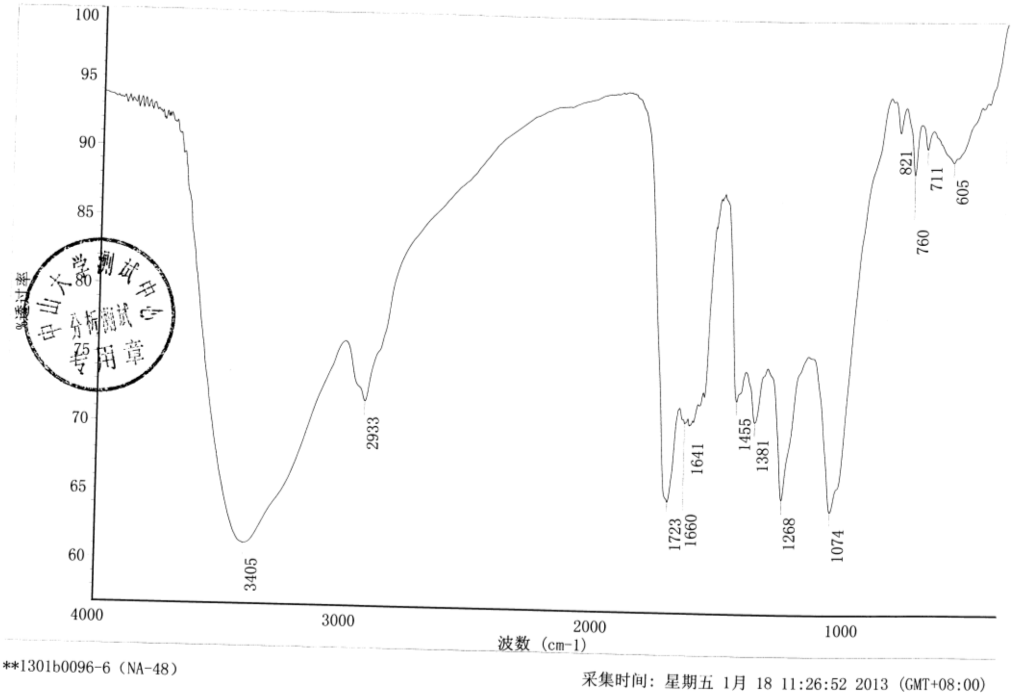

S1. Enzyme Inhibitory Assay

S1.1. Cathepsin B Activity Assay

The assay was performed in 96-well plate according to a published method with modification. Brifely, 50 μL reaction buffer (100 mM sodium acetate (pH 5.5), 1 mM EDTA, 4 mM dithiothreitol) containing 0.0025 unit of cathepsin B from human liver (Sigma, one unit will liberate 1 nanomole of 7-amino-4-methylcoumarin from *Z*-Arg-Arg 7-amido-4-methylcoumarin per min at pH 6.0 at 40 ℃) and 2 μL test compounds dissolved in DMSO were added to each well of a 96-well plate. After preincubation for 15 min at room temperature, 50 μL of reaction buffer containing 100 μM *Z*-Arg-Arg-7-amido-4-methylcoumarin (Sigma) was added and incubated for 30 min at room temperature. Fluorescence was measured using a microplate reader (Wallac 1420 Victor 2, PerkinElmer, HolIand) with an excitation of 355 nm and emission at 460 nm.

S1.2. IMPDH Enzyme Assay

His-taged human IMPDH Π was recombinantly expressed in *Escherichia coli* and purified by Ni-NTA affinity chromotagraphy as described previously. The IMPDH activity assay was performed in 200-μL assay volume of 96-well plate. Briefly, 2 μL of test compound (dissolved in DMSO) or DMSO and 150 μL enzyme buffer containing 100 mM KH_2PO_4, 0.5 mM EDTA pH 8, and 2 mM dithiothreitol and 50 nM IMPDH were added into the plate and incubated at 37 ℃ for 15 min. The reaction was initiated by adding 50 μL reaction buffer containing a final concentration of 200 μM inosine 5-monophosphate (Sigma) and 200 μM NAD (Sigma). The OD was read at 340 nm after incubation at 37 ℃ for 30 min with a microplate reader (Wallac 1420 Victor 2, PerkinElmer, HolIand).

S1.3. PTP1B and SHP2 Activity Assays

Human recombinant PTP1B and SHP2 were expressed in *E. coli* and purifed by Ni-NTA affinity chromotagraphy in our laboratory, respectively. The enzyme activity was measured using p-nitrophenyl phosphate (pNPP) as substrate in a 96-well plate. Brifely, purified recombinant PTP1B or SHP2 (0.05 μg) in 50 μL buffer containing 50 mM citrate (pH 6.0), 0.1 M NaCl, 1 mM EDTA, and 1 mM dithiothreitol (DTT) and test compounds were added to each well of a 96-well plate. After preincubation for 15 min at room temperature, 50 μL of reaction buffer containing 2 mM pNPP was added and incubated at 37 ℃ for 30 min. The PTP1B or SHP2 activity was measured by detecting the absorbance at 405 nm for the amount of produced p-nitrophenol.

S2. Larval Settlement Bioassays

Antifouling activity of compounds was evaluated in settlement inhibition assays with laboratory-reared *B. neritina* larvae. Larval settlement bioassays were performed using sterile 24-well polystyrene plates as previously reported. Briefly, the stock solution of tested samples in DMSO was diluted with autoclaved filtered sea water (FSW) to concentrations ranging from 1 to 300 ppm. Then the EC_{50} and LC_{50} values of active compounds were calculated. In this way, about 20 competent larvae were added to each well in 1 mL of the test solution. Wells containing only FSW with DMSO served as the controls. Three replicates of each treatment were used. The plates were incubated at 27 ℃ for 1 h. The percentage of larval settlement was determined by counting the settled, live individuals under a dissecting microscope and expressing the result as a proportion of the total number of larvae in the well. EC_{50} (inhibits 50% of settlement of *B. neritina* larvae in comparison with the control) and LC_{50} (refers to the concentration that kills 50% of the test organisms in comparison with the control) levels of active compounds were calculated by using the Excel software program.
